# Overexpression *VaPYL9* improves cold tolerance in tomato by regulating key genes in hormone signaling and antioxidant enzyme

**DOI:** 10.1186/s12870-022-03704-8

**Published:** 2022-07-15

**Authors:** Guojie Nai, Guoping Liang, Weifeng Ma, Shixiong Lu, Yanmei Li, Huimin Gou, Lili Guo, Baihong Chen, Juan Mao

**Affiliations:** College of Horticulture, Gansu Agricultural University, Lanzhou, 730070 People’s Republic of China

**Keywords:** Abscisic acid, Overexpression *VaPYL9*, Cold stress, RNA-seq, Expression analysis

## Abstract

**Background:**

Abscisic acid (ABA) has been reported in controlling plant growth and development, and particularly dominates a role in resistance to abiotic stress. The Pyrabactin Resistance1/PYR1-Like /Regulatory Components of ABA receptors (PYR1/PYL/RCAR) gene family, of which the *PYL9* is a positive regulator related to stress response in ABA signaling transduction. Although the family has been identified in grape, detailed *VaPYL9* function in cold stress remains unknown.

**Results:**

In order to explore the cold tolerance mechanism in grape, *VaPYL9* was cloned from *Vitis amurensis*. The subcellular localization showed that *VaPYL9* was mainly expressed in the plasma membrane. Yeast two-hybrid (Y2H) showed *VaPCMT* might be a potential interaction protein of *VaPYL9*. Through the overexpression of *VaPYL9* in tomatoes, results indicated transgenic plants had higher antioxidant enzyme activities and proline content, lower malondialdehyde (MDA) and H_2_O_2_ content, and improving the ability to scavenge reactive oxygen species than wild-type (WT). Additionally, ABA content and the ratio of ABA/IAA kept a higher level than WT. Quantitative real-time PCR (qRT-PCR) showed that *VaPYL9, SlNCED3, SlABI5,* and antioxidant enzyme genes (*POD*, *SOD*, *CAT*) were up-regulated in transgenic tomatoes. Transcriptome sequencing (RNA-seq) found that *VaPYL9* overexpression caused the upregulation of key genes *PYR/PYL*, *PYL4*, *MAPK17/18, *and *WRKY* in transgenic tomatoes under cold stress.

**Conclusion:**

Overexpression *VaPYL9* enhances cold resistance of transgenic tomatoes mediated by improving antioxidant enzymes activity, reducing membrane damages, and regulating key genes in plant hormones signaling and antioxidant enzymes.

**Supplementary Information:**

The online version contains supplementary material available at 10.1186/s12870-022-03704-8.

## Background

As sessile organism, plant can’t move randomly when they are subjected to various adverse environments such as soil salinity, drought, and extreme temperatures. Therefore, they must cope with these abiotic stress factors to adapt to changing environments by various mechanisms [1]. Adverse environmental factors affect plant growth that ultimately causes losses in crop quality and yield all over the world [2, 3]. Although plant has formed a complex response regulation networking to combat stress challenges, it has certain limitations. Therefore, transgenic engineering and transcriptome sequencing are employed to improve adversity stress and enhance plant tolerance [2, 4, 5]. Cold stress is one of the primary environmental factors for plant growth, and cold stress responses and the generation of cold tolerance plants have received much attention in recent years [6]. A full understanding of the molecular mechanisms of cold tolerance will be conducive to plant breeding of cold tolerance species and provide cold tolerance candidate genes for the following researches.

ABA is an important phytohormone that regulates plant growth and development including cell elongation, seed dormancy, leaf senescence, fruit abscission, and stomatal closure [7, 8]. In addition, ABA is also an important stress response phytohormone, and it’s called as a ‘stress hormone’. Many researches have verified that ABA functions as an endogenous messenger in abiotic stress responses, especially in drought, salinity and cold stresses, and plants keep a high ABA accumulation [9–13]. The PYR/PYL/RCAR proteins are currently considered as the typical ABA receptors according to structural, biochemical, and genetic evidences implemented by two independent research groups [10, 11, 14, 15]. The PYR/PYL/RCAR proteins belong to Bet v 1-like superfamily members and include special STAR-RELATED LIPID-TRANSFER (START) related to metabolism, and hydrophobic compounds such as lipids, hormones, and antibiotics [16, 17]. A number of studies found that PYR/PYL/RCAR which was bound to ABA negatively regulated type 2C protein phosphates (PP2C) in ABA signaling transduction, in turn which negatively regulated SNF1-related protein kinase 2 (SnRK2). These three core components formed a double negative regulatory system in ABA signal transduction. Under normal growth conditions, PP2C inactivated SnRK2 through dephosphorylation, and ABA signal was non-activation. When plants were subjected to adverse environmental conditions, ABA production maintained a high level, ABA bound to PYR/PYL/RCAR proteins and promoted interaction function with PP2C to inhibit PP2C phosphatase activity, relieving the inhibition of PP2C on the protein kinase SnRK2, and SnRK2 was activated, and then phosphorylated downstream transcription factors or membrane proteins to arouse ABA signaling reaction [18, 19].

There existed 14 PYL gene members in *Arabidopsis thaliana*, except *PYL13,* other gene sequences and structures were highly conserved, and could bind to ABA inhibiting *PP2C* enzyme activity [11, 20]. Nine of the 14 PYR/PYL/RCAR family members were the most robustly co-purified proteins with *ABI1*. The data supported that *ABI1* interacted with multiple members of the PYR/PYL/RCAR family in *Arabidopsis* [21]. The knockout of *AtPYL8* reduced the sensitivity of ABA on primary root growth and lateral root formation and the overexpression *AtPYL9* promoted the lateral root elongation in the presence of ABA. *AtPYL9* was found to interact with *MYB77* and *MYB44* in *vivo* to regulate auxin-responsive genes affecting roots growth [22, 23]. Additionally, overexpression *SlPYL9* accelerated tomato fruits ripening [24]. PYR/PYL receptors regulated stomatal aperture highlights the relevance of the PYR/PYL pathway to cope with drought stress [14]. PYR/PYL/RCAR ABA receptors were close to abiotic stresses, *RCAR12* and *RCAR13* were confirmed to play positive roles in regulating extreme temperature, including cold and high temperature in *Arabidopsis* [25]. ABA receptors, *RCAR11*, *RCAR12*, *RCAR13* and *RCAR14* showed a hypersensitivity for ABA, increased genes expression related stress responses, as well as enhanced drought tolerance [26]. Under drought and low temperature treatments, the ectopic expression of *OsPYL3* could enhance the cold and drought resistance of *Arabidopsis thaliana*, and *OsPYL10* overexpression had the potential ability to improve both drought and cold stress tolerance of rice [27, 28]. It has been reported that *RCAR5/PYL11* was involved in response to cold stress by delaying seed germination and controlling stomatal closure via ABA-dependent and ABA-independent pathways, respectively [29]. Constitutive expression of *PtPYRL1* and *PtPYRL5* significantly enhanced the resistance to drought, osmotic, and cold stresses by positively regulating ABA signaling in *Populus* L. [30]. Overexpression *AtPYL5* promoted drought tolerance [31, 32]. Exogenous hormones induced *AtPYL8* expression to improve drought stress [33]. *MdPYL9* improved tolerance to drought stress in transgenic apple [34]. The expression level of *GhPYL9-11A* was higher in drought-tolerant cotton cultivars than in drought-sensitive cottons under drought treatment [35]. These functional achievements of PYR/PYL/RCAR showed that researches on stresses tolerance of PYR/PYL/RCAR mainly focused on drought. Although cold stress of PYL genes has been studied in several species, its knowledge was less than drought.

To date, a total of eight *VvPYL* genes were identified from the grape genome database, which could respond to cold, salt, and PEG stresses. The researcher successfully cloned six *PYL* genes from the wild Chinese V. yeshanensis (‘Yanshan-1’), and overexpression *VyPYL9* in *Arabidopsis* enhanced ABA sensitivity, and improved drought tolerance under ABA and drought treatments [36, 37]. *VvPYL1* modulated ABA signaling pathway by inhibiting PP2C expression [38]. *VlPYL1* from ‘Kyoho’ promoted anthocyanin accumulation in grape berry skin [39]. There were most widely studies on CBF and COR genes related to cold stress in grape [40, 41]. However, knowledge of *PYLs* related to cold stress is relatively narrow in grape. Therefore, in this study, *VaPYL9* was cloned from *Vitis Amurensis* with strong cold tolerance, and it was ectopically expressed in tomatoes. Results showed that the *VaPYL9* overexpression was induced by cold stress. The overexpression of *VaPYL9* in tomatoes contributed to an increasing of the ability to scavenge reactive oxygen species and reduce membrane damage, and keep a high ABA level in transgenic tomatoes. Through RNA-seq, the result showed *VaPYL9* bound to ABA leading to a series of reactions in metabolism and biosynthesis, and mainly focused on MAPK and plant hormones signaling transduction paths under cold stress. In all, these findings indicate that *VaPYL9* dominates a positive role in plant response to cold stress and may be an important candidate gene for molecular breeding for the improvement of stress tolerance in grape.

## Results

### Detailed information and evolutionary analysis of *VaPYL9*

**Fig. 1:**
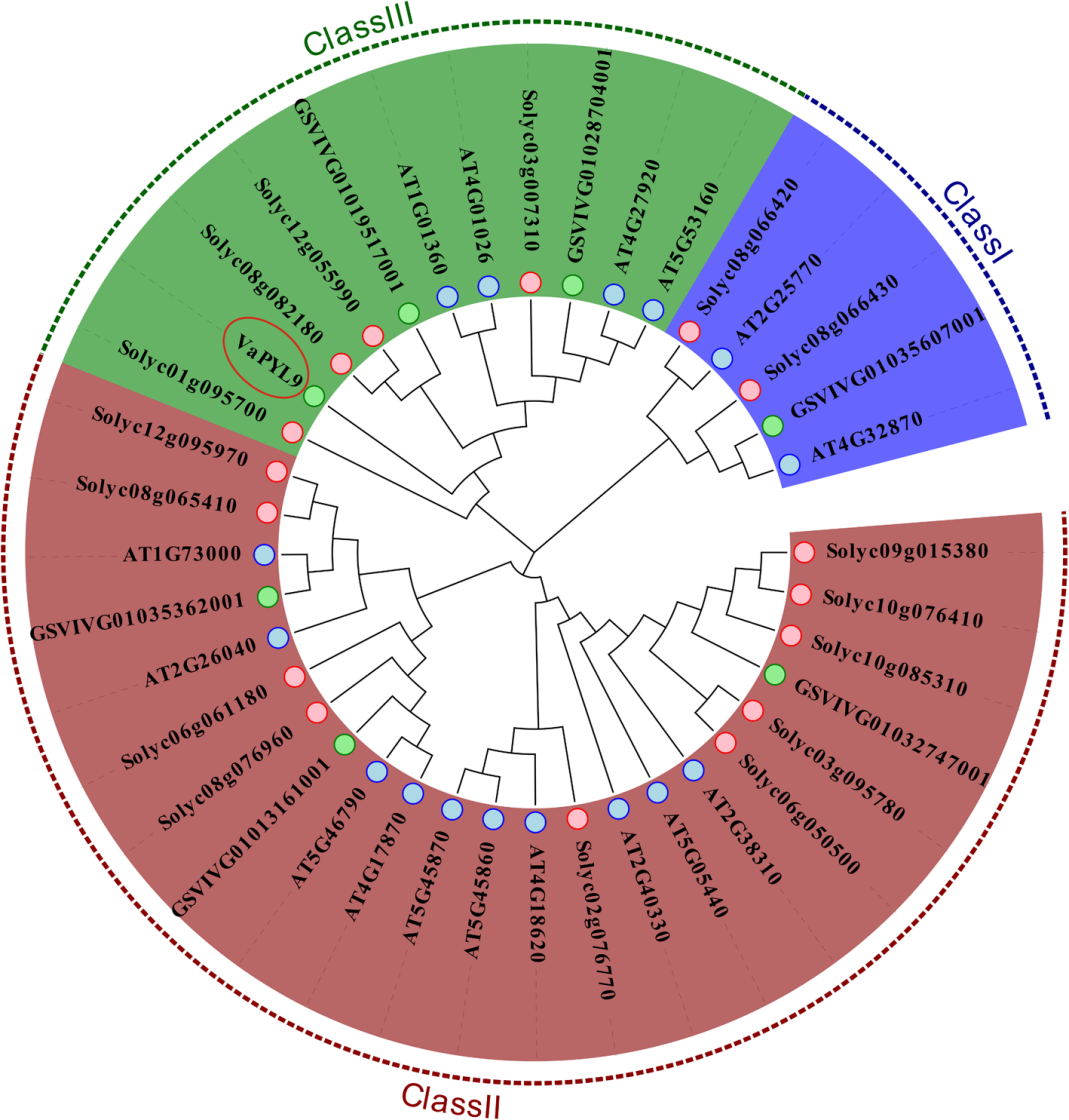
Evolution analysis of PYL genes from grape, *Arabidopsis thaliana*, and tomato. The phylogenetic tree was constructed using PYL sequences of grape, tomato, and *A. thaliana* in MEGA X*,* which was clustered into three subgroups. Different background colors represent different subgroups. *VaPYL9* marked with red ellipse was in the III class. The green circle, blue circle, and pink circle represent grapes, *A. thaliana*, and tomato PYL genes, respectively

In order to have a basic understanding of *VaPYL9* (GSVIVG01027078001), the physicochemical property was analyzed in supplementary Table S1. The online prediction found that isoelectric point (PI) was 6.38, the molecular weight was 20.05 KD, grand average of hydropathicity (GRAVY) was -0.193, instability index(II) was 37.65, and the aliphatic index(AI) was 95.67. The subcellular localization prediction showed that *VaPYL9* was expressed mainly in cytoplasm. VaPYL9 protein was stable because instability index was less than 40. Protein was hydrophilic amino acid when GRAVY was negative value. Therefore, VaPYL9 protein had strong hydrophilicity. Phylogenetic tree analysis showed that PYL genes from grape, tomato, and *Arabidopsis* were divided into three subgroups, and *VaPYL9* was clustered in III subgroup (Fig. 1). *VaPYL9* and most tomato genes were clustered in a close evolutionary branch. Multiple sequences alignment showed that PYL genes had a polyketide cyclase/dehydrase and lipid transport domain (polyketide-cyc2, Pfam accession: PF10604) and highly conservative amino acid sequences (Supplementary Fig. S1). The finding indicated *VaPYL9* might have a similar function to PYL genes of tomatoes.

### Transient expression of *VaPYL9* in tobacco

**Fig. 2:**
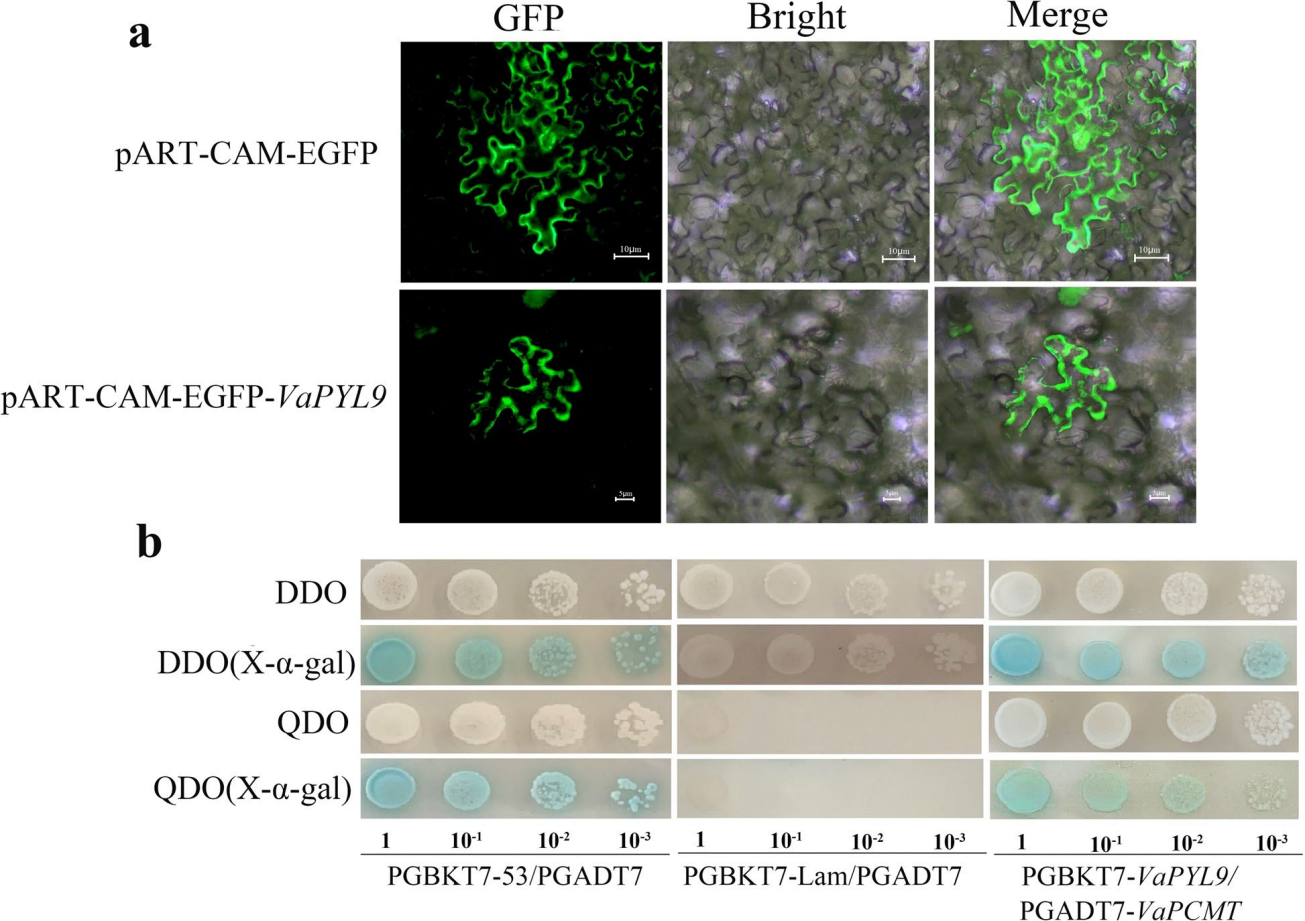
(**a**). Subcellular localization of *VaPYL9*. Four-weekend tobaccos were infected with bacterial liquid. After 24 h, infected leaves were observed and photoed in cell membrane and cytoplasm. (**b**). Yeast two -hybrid (Y2H) assay. Combinations of pGADT7-T with pGBKT7-53 and pGBKT7-Lam were served as positive and negative controls, respectively. *VaPYL9* and *VaPCMT* were commonly transformed into Y2H gold. The result indicated that there was an interaction relationship between *VaPYL9* and *VaPCMT* Proteins

Based on the above online prediction, *VaPYL9* was located mainly in the cytoplasm. To further verify the prediction result, the *pART-CAM-EGFP-VaPYL9* vector was constructed. The *pART-CAM-EGFP-VaPYL9* and pART-CAM-EGFP empty construct plasmids were transformed into *Agrobacterium tumefaciens* strain GV3101 (Supplementary Fig. S2), and then were injected into tobacco leaves. The physical investigation found that *VaPYL9* was not only located in plasma membrane and nucleus but also in cytoplasm (Fig. 2a).

### The cloning of *VaPYL9* and obtaining of transgenic tomatoes

To specifically investigate cold tolerance of the *VaPYL9*, the Open Reading Framework (ORF) with 591 amino acids was inserted into overexpression vector pCAMBIA1301. The presence of *VaPYL9* cDNA fragment was verified by PCR and sequenced in *Escherichia coalition* (DH5α) and *Agrobacterium tumefaciens* strain GV3101 (Supplementary Fig. S3). Through a *Agrobacterium tumefaciens* medicated method, *VaPYL9* fragment was successfully introduced into tomatoes and identified by PCR (Supplementary Fig S4). Three stable and homozygous T3 lines (#2, #6, #9) were screened from transgenic tomato lines and named OE1, OE2, and OE3, respectively.

### The interaction proteins screening of *VaPYL9*

To preliminary investigate interaction proteins of *VaPYL9* from AD library-related cold stress, pGBKT7-*VaPYL9* was constructed and introduced into Y2H gold (Supplementary Fig. S5a). Self-activation and toxicity detection were detected in DDQ and DDQ (x-α-gal) plates. Bacterial plaque didn’t become blue in DDQ and DDQ (x-α-gal) plates. This indicated that *VaPYL9* had no self-activation and toxicity (Supplementary Fig. S6a). Through the mating hybridization method, interaction proteins of *VaPYL9* were preyed from AD library related-cold stress. The presence of potential interaction proteins was verified by PCR using AD vector primers. Finally, *VaPCMT* (protein-L-isoaspartate O-methyltransferase 1) was screened by blast in NCBI. pGADT7-*VaPCMT* was commonly constructed and introduced into Y2H gold (Supplementary Fig. S5b). Using the same procedure, self-activation and toxicity were detected, indicating that *VaPCMT* didn’t produce self-activation and toxicity (Supplementary Fig. S6b). pGBKT7-*VaPYL9* and pGADT7-*VaPCMT* proteins were co-transformation into Y2H gold in QDO and QDO(x-α-gal) plates (Supplementary Fig. S7). Compared to negative and positive controls, bacterial plaque became gradually blue in DDQ and DDQ (x-α-gal) plates after 2 days. *VaPCMT* was a potential interaction protein of *VaPYL9*. Comprehensive above results, the findings showed that *VaPYL9* and *VaPCMT* might be a potential interaction function in *vivo* (Fig. 2b).

### Phenotype observation and degree of membrane damage in tomatoes under cold stress

**Fig. 3:**
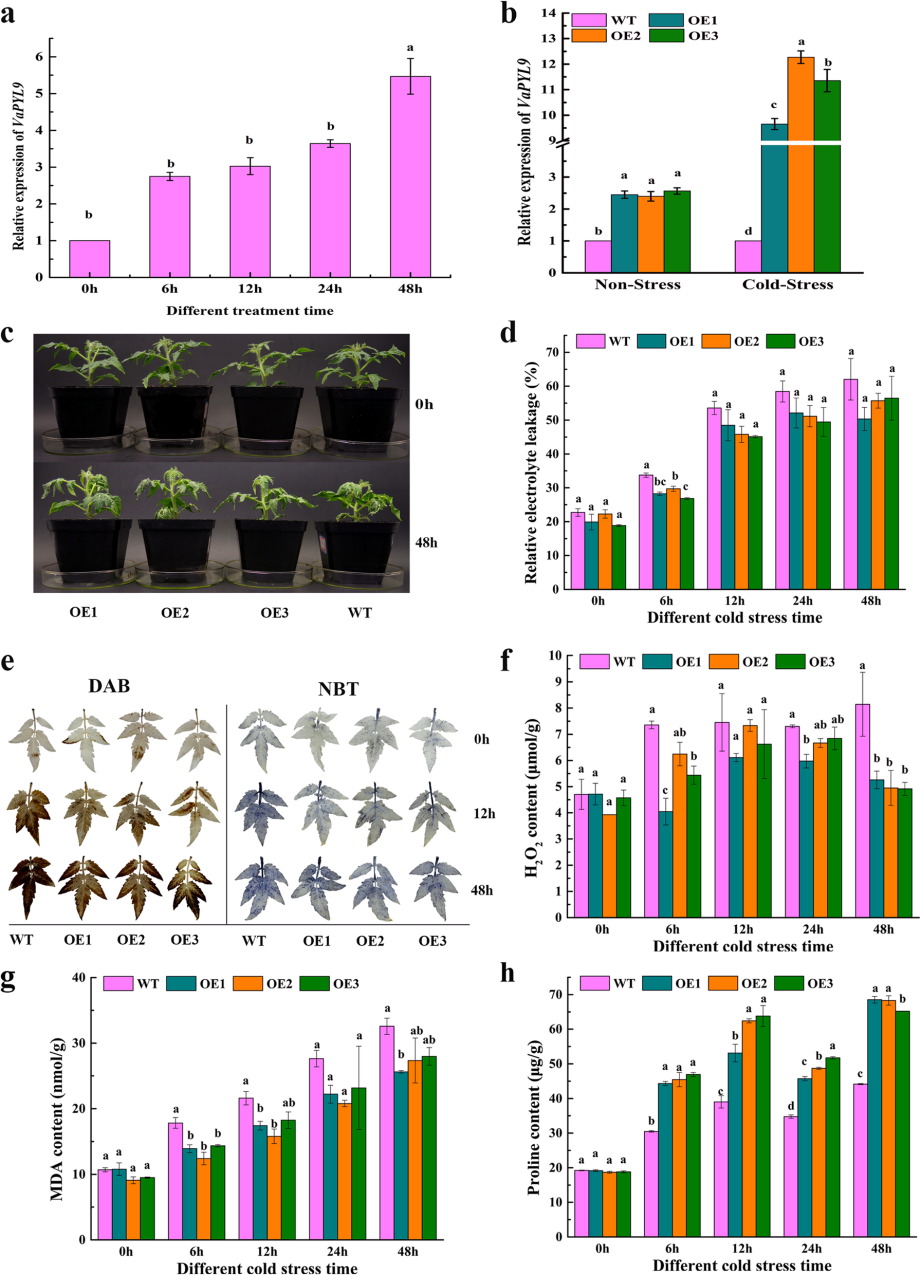
Phenotype investigation and degree of membrane damage between WT and OE. Six-week-old tomatoes were used to detect physiological indexes related to cold stress. (**a**). Expression level of *VaPYL9* in grape under 4 ℃ stress for 0 h, 6 h, 12 h, 24 h, 48 h. (**b**). Expression level of *VaPYL9* in WT and transgenic tomatoes under cold stress and nonstress. (**c**). Tomatoes phenotype was observed in WT and OE under 4 ℃ for 0 h, 48 h. (**d**). The determination of relative electrolyte leakage in WT and OE plants under 4 ℃ for 0 h, 6 h, 12 h, 24 h, 48 h. (**e**). The DAB and NBT staining of WT and OE plants under 4 ℃ for 0 h, 12 h, 48 h. (**f**). The content of H_2_O_2_ was determined in WT and OE plants under 4 ℃ for 0 h, 6 h, 12 h, 24 h, 48 h. (**g**). The content of MDA was determined in WT and OE plants under 4 ℃ for 0 h, 6 h, 12 h, 24 h, 48 h. (**h**). The content of proline was determined in WT and OE plants under 4 ℃ for 0 h, 6 h, 12 h, 24 h, 48 h. The bar represents the value of standard error (*p* < 0.01). Lowercase letters represent the significance level. Means and SE values were calculated from at least three independent experiments

To evaluate the role of *VaPYL9* in response to cold stress, WT and OE lines were exposed to a long-term cold environment. qRT-PCR showed that relative expression of *VaPYL9* was upregulated when grapes were induced by cold stress, and up to a maximum in 48 h (Fig. 3a). Compared with WT tomatoes, transgenic tomatoes were highly expressed in cold stress (Fig. 3b). Through phenotype observation in WT and OE lines under 4 ℃ cold stress for 0 h, 48 h, results indicated that WT and OE lines showed wilting difference but no significant in appearance, and WT showed cold damage and leaves coiled and wilted more heavily than OE lines (Fig. 3c). Some indexes involved in membrane damages were determined. In general, REL is frequently associated with abiotic stress, the membrane permeability tends to the increasing of REL when plants are exposed to abiotic stress. With the prolongation of a cold duration time, REL showed an increasing trend, indicating the degree of membrane damage was increasing in WT and OE lines. From 12 h, relative electrolyte leakage (REL) was quickly increasing. REL of OE plants was lower than WT under cold stress (Fig. 3d). The result showed *VaPYL9* overexpression enhanced membrane protection feature.

DAB and NBT staining were used to evaluate H_2_O_2_ and O^−2^ content at 0 h, 12 h, 48 h. Leaves staining showed there was no significant colour difference in WT and OE lines under non-stress. H_2_O_2_ and O^−2^ didn’t accumulate and kept a homeostasis in leaves. Under cold stress, the degree and area of leaves staining in WT were deeper and expanded than OE lines, respectively (Fig. 3e). WT plants hold a high accumulation of H_2_O_2_ and O^−2^ under cold stress for 48 h. As was shown in Fig. 3f, through the determination of H_2_O_2_, result found H_2_O_2_ content of OE was lower than WT at all time points, and OE was lowest at 48 h. The data supported that OE plants had prominent scavenging effects against superoxide anion radicals. *VaPYL9* expression was related to oxidative stress responses. Malondialdehyde (MDA) content showed an increasing trend in WT and OE with the time of cold stress increasing, but in all time points MDA of OE was lower than WT lines. Low-level MDA indicated that lipid peroxidation of membrane was relatively low (Fig. 3g). There were almost no differences in proline content between OE and WT under non-stress. Proline content of OE was higher than WT and reached a maximum at 48 h. High-level proline protected plant cells from cold stress (Fig. 3h). Taken together, the overexpression *VaPYL9* improved the ability to scavenge reactive oxygen species (ROS), and reduce the degree of membrane lipid peroxidation and cell dehydration property to protect tomatoes from cold stress.

### The changing of antioxidant enzymes activity and endogenous hormones level under cold stress

**Fig.4:**
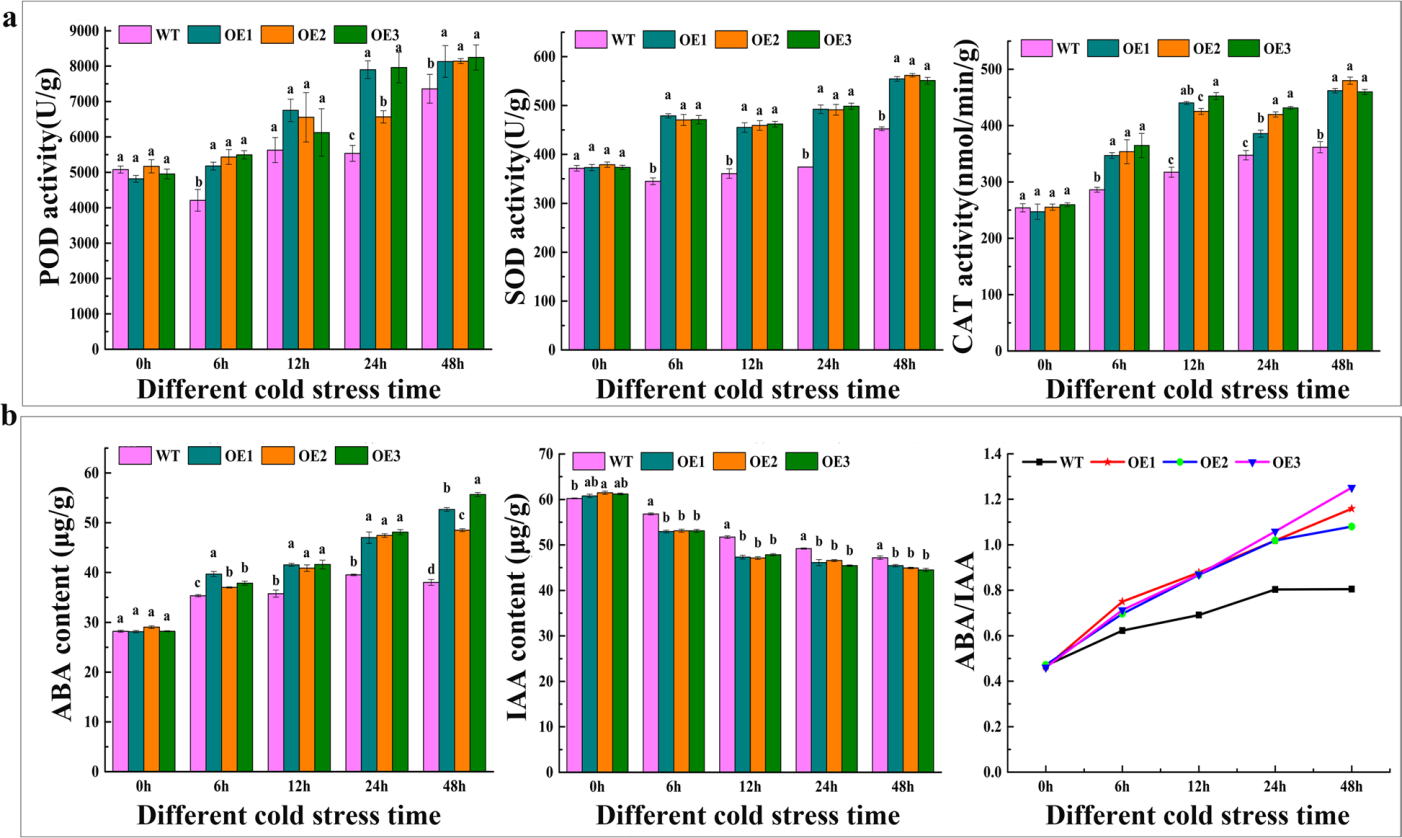
The changing of antioxidant enzyme activities and endogenous hormones in WT and OE plants. Six-weekend tomatoes were used as materials to detect enzyme activities and endogenous hormones. (**a**). POD, SOD, and CAT enzymes activities were determined in WT and OE plants under 4 ℃ for 0 h, 6 h, 12 h, 24 h, 48 h. b. (**b**). The content of IAA and ABA was determined, and ABA/IAA was calculated in WT and OE plants under 4 ℃ for 0 h, 6 h, 12 h, 24 h, 48 h. The bar represents the value of standard error (*p* < 0.01). Lowercase letters represent the significance level. Means and SE values were calculated from at least three independent experiments

Catalase (CAT), superoxide dismutase (SOD), and peroxidase (POD) play an important role in maintaining the homeostasis of ROS metabolism and antioxidation defense system. The results showed that there was no significant difference in enzyme activities of OE and WT at 0 h, while CAT and POD activities of WT and OE lines showed an increasing trend in all cold time points. Interestingly, *VaPYL9* expression tomatoes exhibited higher activities of SOD, CAT, and POD enzymes compared to WT under cold stress. These three enzyme activities reached a maximum under cold stress for 48 h (Fig. 4a). In all, the results indicated that *VaPYL9* expression caused an increase of antioxidant enzymes activity, reducing ROS damage for tomatoes.

Endogenous hormones were determined by High Performance Liquid Chromatography (HPLC). The content of ABA was increasing while IAA was decreasing with the prolongation of cold duration time under cold stress. *VaPYL9* expression tomatoes exhibited higher content of ABA compared to WT under cold stress, and ABA content reached a maximum under cold stress for 48 h. On the contrary, *VaPYL9* overexpression tomatoes exhibited lower content of IAA compared to WT under cold stress. ABA/IAA of OE was gradually increasing compared to WT with cold duration time increasing (Fig. 4b). The findings supported that *VaPYL9* overexpression resulted in higher ABA content and ABA/IAA, promoting cold tolerance for plants.

### qRT-PCR analysis

**Fig. 5:**
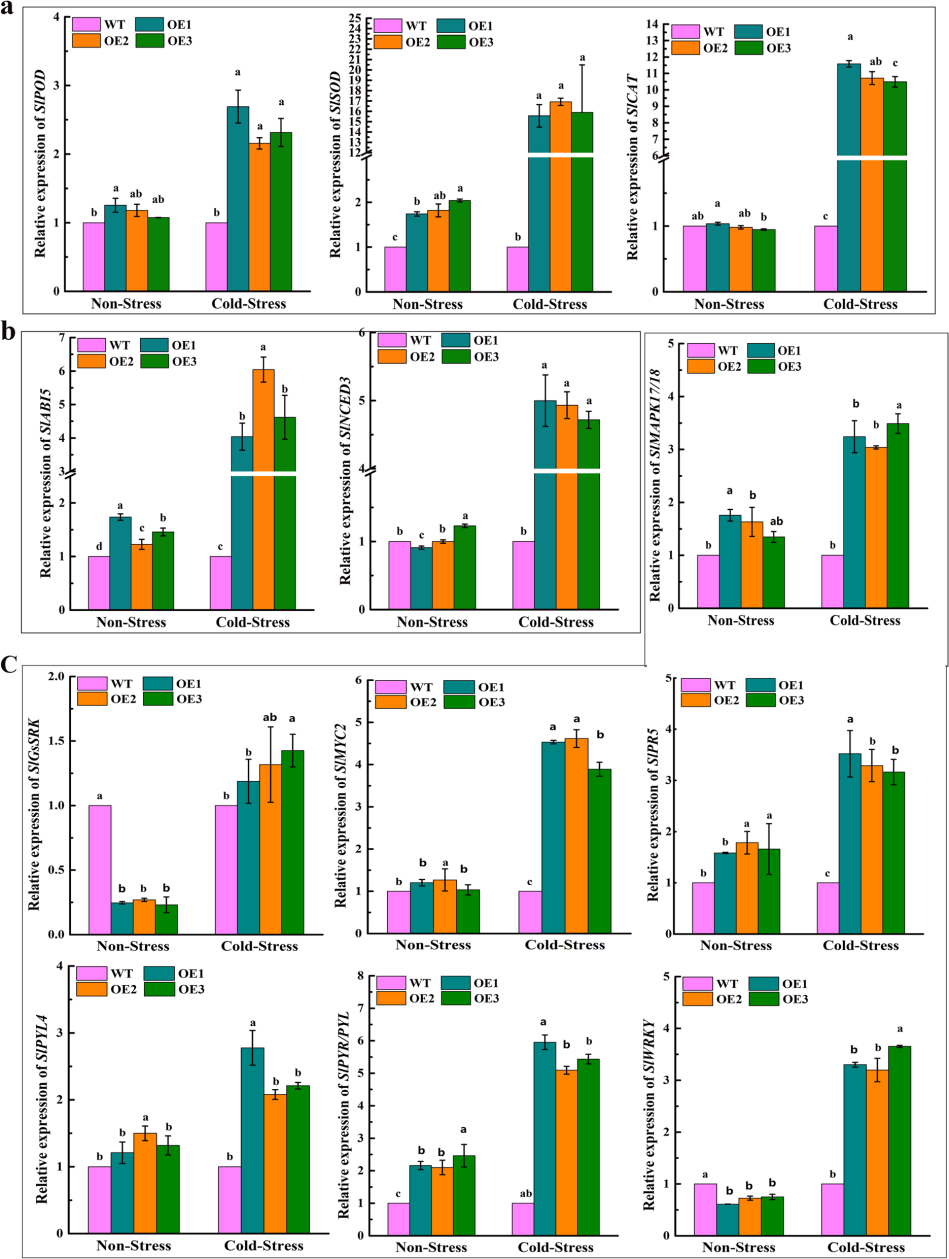
qRT-PCR analysis. (**a**). Expression level detection of key enzyme genes related to *POD*, *SOD*, and *CAT* genes under 4 ℃ for 0 h, 48 h. (**b**). Expression level detection of *NCED3* and *ABI5* genes related to ABA path under 4 ℃ for 0 h, 48 h. (**c**). Expression level detection of *MAPK17/18*, *GsSRK*, *MYC2*, *PR5*, *PYL4*, *PYR/PYL*, *WRKY* key genes in RNA-seq. The bar represents the value of standard error (*p* < 0.01). Lowercase letters represent significance level. Means and SE values were calculated from at least three independent experiments

qRT-PCR was employed to perform expression level measurement under cold stress for 0 h, 48 h. The expressions of *SOD* (NM_001311084.1), *CAT* (NM_001247257), and *POD* (NM_001302921.2) key genes involved in antioxidant enzymes were significantly upregulated under cold stress. High expresssion levels of antioxidant enzymes led to the increase of SOD, CAT, and POD activities (Fig. 5a). Compared to WT, *SlNCED3* (Solyc07g056570.1), a key rate-limiting enzyme gene in the process of ABA synthesizes, and *SlABI5* (XM_010327616.3), a key gene in ABA signaling transduction was up-regulated in OE lines than WT under cold stress (Fig. 5b). Altogether, these findings indicated that *VaPYL9* gene was highly expressed in transgenic plants when plants were subjected to cold stimulation, and *VaPYL9* gene could positively respond to cold stress.

### Transcriptome sequencing analysis

**Fig. 6:**
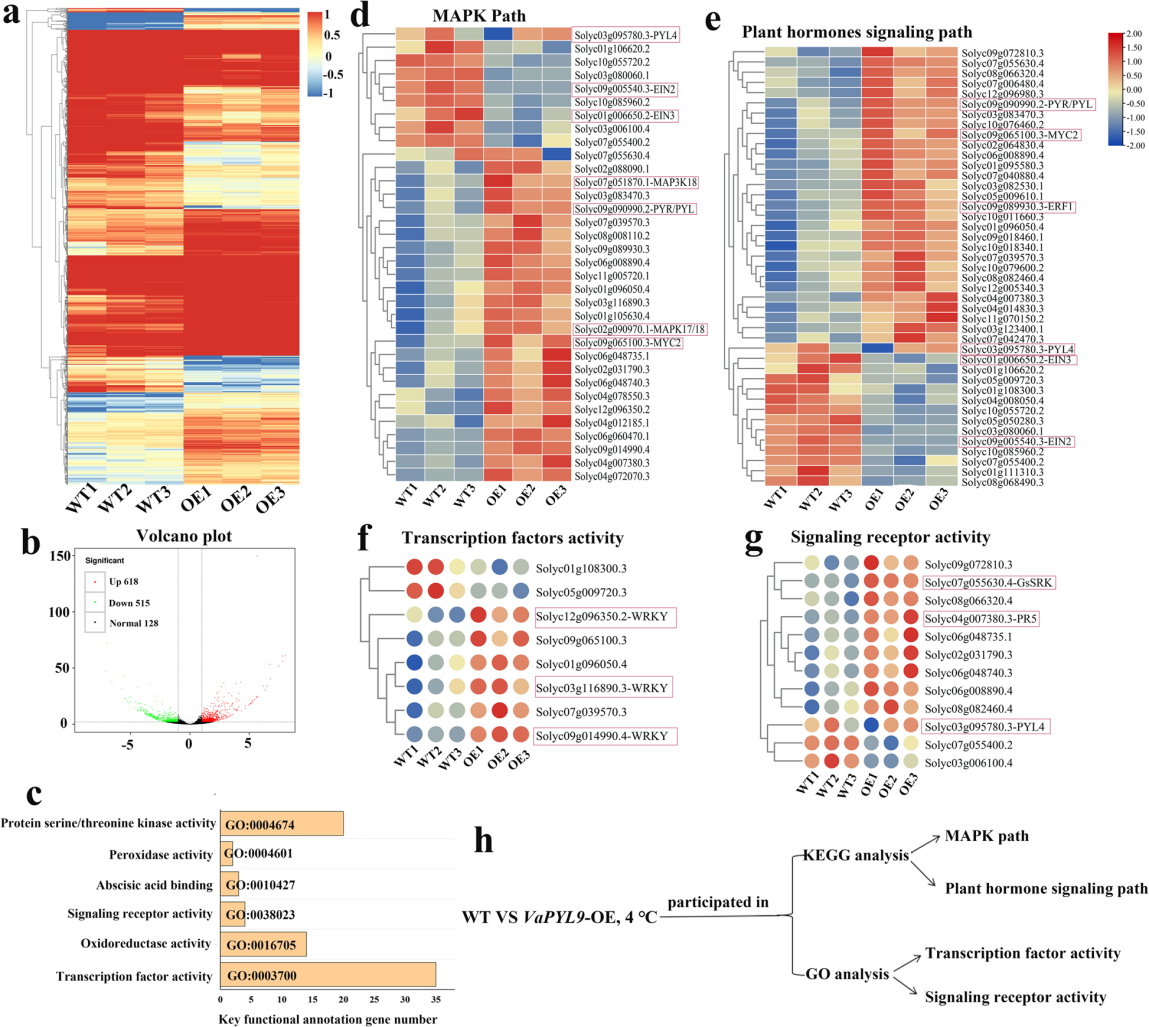
The analysis of transcriptome sequencing in WT and OE plants under 4 ℃ for 48 h. (**a**) The heatmap of total differential expression genes. Rectangles in red and blue represent up and down-regulated expression. (**b**) The number of DEGs was shown by the volcano plot. Dots in red, green and red represent up, down and normal regulated genes, respectively. The number of the dots indicates the number of DEG. (**c**) The number of DEGs in main GO enrichments. (**d**) The expression level of key differential expression genes in MAPK path. Rectangles in red and blue represent up and down-regulated expression. (**e**) Expression level of differential expression genes in plant hormone signaling path. Rectangles in red and blue represent up and down-regulated expression. (**f**) Key differential expression genes expression in transcription factors activity. Rectangles in red and blue represent up and down-regulated expression. (**g**) Key differential expression genes expression in signaling receptor activity. Rectangles in red and blue represent up and down-regulated expression. (**h**) *VaPYL9* overexpression participated in MAPK and plant hormone signaling KEGG path, and improved the activity of transcription factors and signaling receptors

To investigate the role of *VaPYL9* gene in regulating cold tolerance, the RNA-Seq analysis was performed using the six-week-old transgenic and WT tomato seedlings incubated at 4 ℃ for 48 h and identified differentially expressed genes (DEGs). Basic information of sequencing data was listed in Table 1, a total of 44 Gb clean data was gained, GC content of every sample was up to over 40%, and base percentage Q30 of every sample was over 94.43%. According to the statistics of the comparison results, the comparison efficiency between reads of each sample and reference genome ranged from 95.00% to 95.97%. A total of 1270 DEGs were found in WT vs OE lines subset based on the criteria fold change > 2 and *P* < 0.05, of which 740 DEGs (*VaPYL9*-activated genes) were up-regulated, 530 DEGs were down-regulated (*VaPYL9*-repressed genes) (Fig. 6a, b). GO (Gene Ontology) showed that key functional annotations were associated with transcription factor activity, oxidoreductase activity, signaling receptor activity, abscisic acid binding, peroxidase activity, and protein serine/threonine kinase activity (Fig. 6c). KEGG analysis found that *VaPYL9* overexpression mediated MAPK and plant signaling transduction paths to regulate cold tolerance. There were 34 DEGs in MAPK path, of which 25 DEGs were up-regulated in OE compared to WT (Fig. 6d). MAPK activated the upstream ABA signaling to function in cold defense, *MAPK17/18* was highly expressed in adapting to stress (Fig. 6d). There were 43 DEGs in plant signaling transduction path, of which 29 DEGs were up-regulated in OE compared to WT (Fig. 6e). Key *PYR/PYL* and *MYC2* genes were up-regulated in OE compared to WT. Transcription factors mainly were involved in *WRKY*, and were up-regulated in OE lines than WT (Fig. 6f). Signaling receptor activity expression was higher than WT, mainly involved in *PYL4*, *PR5* and *GsSRK* (Fig. 6g). Through qRT-PCR verification, *MAPK17/18, PYR/PYL*, *MYC2, WRKY, PYL4*, *PR5* and *GsSRK*, some key genes involved in MAPK, plant signaling transduction, signaling receptor activity, and transcription factors activity were up-regulated in transgenic under cold stress (Fig. 5c). In summary, these data demonstrated that *VaPYL9* played a positive role in cold stress tolerance in tomatoes mediated by MAPK and plant signaling transduction paths, improving transcription factors and signaling receptor activity (Fig. 6h).

**Table 1:** Detailed information of RNA-seq data

Index Samples		WT1	WT2	WT3	OE1	OE2	OE3
Sequencing data statistics	Clean reads	20,112,132	27,809,393	25,022,722	25,669,474	25,182,500	23,361,247
	Clean bases	6,011,496,948	8,314,175,984	7,490,345,612	7,672,294,656	7,525,008,224	6,982,459,062
	GC Content	42.66%	42.07%	42.46%	41.79%	41.77%	41.75%
	% ≥ Q30	94.52%	94.46%	94.43%	94.94%	95.01%	94.92%
Based on selected reference genome, statististic of sequence alignment results between sample sequencing data	Total Reads	40,224,264	55,618,786	50,045,444	51,338,948	50,365,000	46,722,494
	Mapped Reads	38,466,134(95.63%)	53,225,213(95.70%)	48,026,349(95.97%)	48,812,363(95.8%)	47,848,224(95.00%)	44,748,280(95.77%)
	Uniq Mapped Reads	35,062,965(87.17%)	50,662,268(91.09%)	46,241,168(92.40%)	47,515,584(92.55%)	46,601,110(92.53%)	43,612,976(93.34%)
	Multiple Map Reads	3,403,169(8.46%)	2,562,945(4.61%)	1,785,181(3.57%)	1,296,779(2.53%)	1,247,114(2.48%)	1,135,304(2.43%)
	Reads Map to ‘ + ’	18,558,770(46.14%)	26,181,517(47.07%)	23,744,948(47.45%)	24,259,222(47.25%)	23,771,258(47.20%)	22,257,873(47.64%)
	ReadsMap to ‘-’	19,022,283(47.29%)	26,451,663(47.56%)	23,917,058(47.79%)	24,327,689(47.39%)	23,850,045(47.35%)	22,312,867(47.76%)

## Discussion

### ABA regulates cold tolerance in plant

Low temperature is a serious threat to crop yield stability, plant growth, quality improvement, and plant geography distribution [42]. To adapt to cold stress, a variety of biochemical and physiological processes have formed in plant. Plants modulate multiple morphological and physiological processes to maintain cellular and metabolic homeostasis, including changes in phytohormone homeostasis [43]. ABA is an important stress hormone that adjusts plant stress such as extreme temperature, salt, drought [1]. ABA has been demonstrated to be involved in the cold stress response through the regulation of a series of specific stress-responsive genes, and ABA can coordinate several phytohormones that integrated with cold signaling to regulate cold stress [13]. In recent years, an increasing number of studies have attached the importance of studying the mechanisms of ABA in cold tolerance. Exogenous ABA can improve the content of proline and soluble sugar, enhance water retention, reduce membrane lipid peroxidation, protect membrane integrity, and reduce photosynthetic characteristics [44, 45]. In addition, exogenous ABA application altered the expression of or related-cold and ABA genes, improving resistance to cold in *Bermudagrass*, maize, and cucumber [46–48]. Here, through previous RNA-seq, ABA receptor *VaPYL9* was selected from DEGs related to cold stress to further verify the function of cold tolerance.

### The ABA receptors PYLs were involved in cold stress

The function of PYL proteins on ABA-mediated stress responses has been well studied [18]. ABA receptors *OsPYL3*, *OsPYL10*, and *AtPYL11* enhanced the cold tolerance of plants [27–29]. *Vitis Amurensis Rupr*, which has strong cold hardiness can withstand -35 ℃ [49]. In this study, *VaPYL9* gene from *Vitis Amurensis Rupr* was cloned, evolutionary analysis showed that *PYLs* could be classified into three subfamilies (Fig. 1). The result was consistent with clusters in *Brassica napusin* and rice [50, 51]. The alignment of amino acid sequences revealed that these *PYLs* were highly conserved (Fig. 1b), especially amino acid residues that involved in ABA binding and PP2C interaction [52]. *VaPYL9* had a polyketide-cyc2 domain related to polyketide cyclase/dehydrase and lipid transport as the same result in the previous study [53]. Most *AtPYLs* were mainly localized to the cytoplasm and the nucleus, and transduced ABA signals to activate a series of responses to various environmental factors [10, 11]. The same location result was found in this study, through predication online and physical transient transformation in tobacco, which showed that the *VaPYL9* was also localized in the cytoplasm and nucleus (Fig. 2a). When plants were subjected to abiotic stress, ABA was synthesized and bound to PYL proteins, and the ABA-PYL complex interacts with PP2Cs to initiate ABA signal transduction [31, 54]. It has been reported that PYLs interacted with specific PP2C members in an ABA-dependent or ABA-independent manner [20]. In the present study, through screening of the Y2H method, *VaPCMT* was identified to be a potential protein with *VaPYL9* commonly to mediate cold tolerance (Fig. 2b), and *PCMT* was related to oxidative stress in the previous study [55].

### Overexpression of *VaPYL9* improved cold tolerance mediated by scavenging reactive oxygen species, reducing membrane damage in tomato

Many previous evidences have shown that PYLs play crucial roles in ABA-mediated responses to cold stress [27–29]. Previous research reported that *AtPYL9*, the homolog sequence of *VaPYL9* in *Arabidopsis*, enhanced ABA sensitivity and drought stress when expressed to high levels in transgenic *Arabidopsis* [56]. But PYL9 function was seldom involved in cold stress compared to drought. To preliminarily explore the function of *VaPYL9* in response to cold in grape, overexpressing-*VaPYL9* tomatoes lines were generated to evaluate the response of these transgenic plants under cold stress.

To have a good understanding of mechanisms associated with *VaPYL9*-induced cold tolerance, changes in several physiological and biochemical characteristics which are known to be associated with response to cold stress were determined. When plants are exposed to abiotic stress leading to an increasing of ROS production, which can cause oxidative damage to cellular components, and the intracellular ROS level is considered as an important indicator of cell death in response to stress conditions [57]. Here, results showed that REL, H_2_O_2_ and MDA accumulation in WT plants was higher and proline was lower than in the transgenic lines after cold stress (Fig. 3d, e, f, g, h), suggesting that *VaPYL9* expression is related to oxidative stress responses. To protect cells and membrane integrity from effects of excessive ROS, plants have evolved a series of complex antioxidant defense mechanisms to keep the homeostasis of ROS metabolism, such as SOD, CAT, and POD [58–60]. The results of the present study showed that *VaPYL9* overexpression, transgenic tomatoes exhibited higher activities of SOD, CAT, and POD enzymes compared to WT under cold stress (Fig. 4a), suggesting that the reducing ROS in transgenic tomatoes may result from increasing ROS metabolism by these enzymes. qRT-PCR showed *SOD*, *CAT* and *POD* gene of OE lines was up-regulated under cold stress compared to WT (Fig. 5a). Coincidentally with plant stress, overexpression of *GhPYL9-11A*, *PtPYL1*, and *PtPYL5* genes also caused an increasing in activities of antioxidant enzymes and the ability to scavenge ROS [30, 35, 61]. *SlNCED3*, a key rate-limiting enzyme gene in the process of ABA synthesizes, and *SlABI5*, a key gene in ABA signaling transduction was up-regulated in OE lines under cold stress compared to WT (Fig. 5b). Consistence with the result that *NCED3* promoted ABA biosynthesis under stress and *ABI5* was related to multiply hormones adapting to various stresses [62, 63]. With the prolongation of a cold duration time, the ratio of ABA/ IAA was increased in OE lines than in WT (Fig. 4b). The result was similar to the previous study, the ratio of ABA/ IAA was increased in sugarcane leaves under cold stress [64]. The result showed that the increasing activities of antioxidant enzymes, reducing membrane damage, improving the ability to scavenge ROS, and high ABA content constitute an important physiological mechanism in plants driven by *VaPYL9* overexpression under cold stress.

### *VaPYL9* improved cold tolerance mediated by MAPK pathway and multiply hormones coordination

Additionally, RNA-seq found a total of 1270 DEGs were found in WT vs OE lines subset, including 740 DEGs (*VaPYL9*-activated genes) that were up-regulated, and 530 DEGs were down-regulated (*VaPYL9*-repressed genes) (Fig. 6a, b). Through the KEGG map, the result showed overexpression *VaPYL9* activated MAPK pathway and ethylene, and ABA commonly promoting adaption to cold stress in plant (Fig. 6d, e). MAPK was induced by ABA during abiotic stress. A combination of MAP3Ks, MAP3K17/18, MAP2K MKK3 and MAPKs MPK1/2/7/14 MAPK cascade related to PYR/PYL/RCAR mediated ABA signaling pathway, MPK1 and MPK2 may also phosphorize many ABA effector proteins [65, 66]. Similarly, *PYR/PYL* and *MAP3K17/18* were up-regulated in transgenic plants under cold stress (Fig. 6d). *PYL6* responded to JA by interacting with *MYC2*. In the presence of ABA, the interaction of the two become a strength between ABA and JA signaling pathways [67]. The ERF1 binding to response element to regulate abiotic stress and mediated ROS signaling to response salt stress [68, 69]. Coincidentally, in this study *MYC2* indirectly regulated *ERF1* to connect JA and ethylene enhancing defending stress, the two were up-regulated in overexpression *VaPYL9* induced by cold stress (Fig. 6d, e). In all, *VaPYL9* response to cold stress probably thereby MAPK and hormone signaling paths.

## Conclusion

**Fig. 7:**
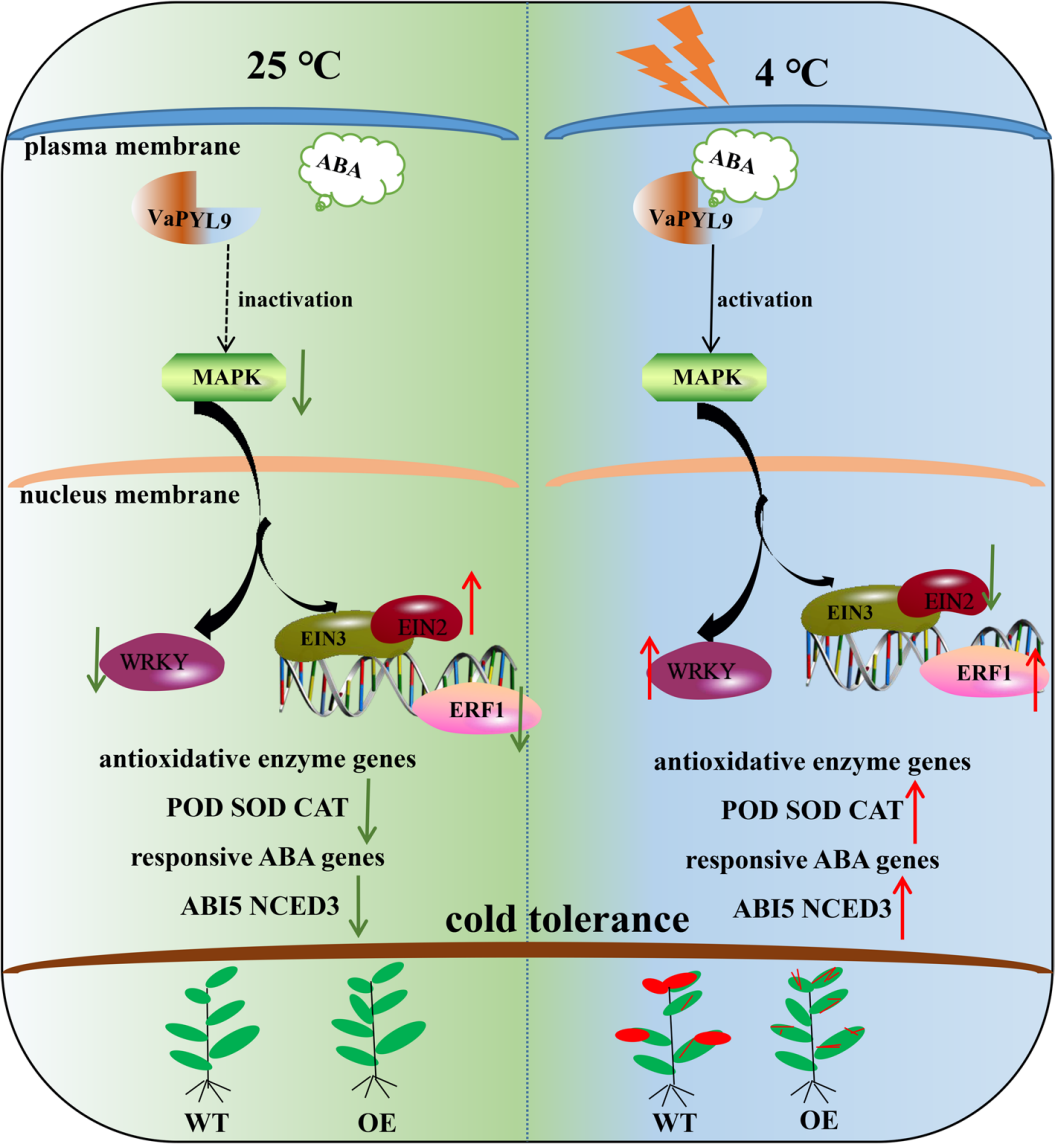
A systematic model of the role of *VaPYL9* in the response of tomatoes under 25 ℃, 4 ℃. Green and red arrows represent down and up-regulated expression levels in WT vs OE, respectively. The size of the red areas represents the degree of leaves damage

In summary, a working model of the role of *VaPYL9* in the response of tomatoes to cold stress was proposed (Fig. 7). *VaPYL9* overexpression induces the high expression of *SlNCED3* and *SlABI5* leading to ABA accumulation. *VaPYL9* might be active in MAPK path of the downstream of ABA signaling response and plant hormones signaling by binding to ABA. The overexpression of *VaPYL9* enhanced cold sensitivity in tomatoes by reducing membrane damage and improving activities of enzymatic antioxidant defense systems. RNA-seq indicated that MAPK and other hormones also might be involved in improving cold response. Our works suggest that the *VaPYL9* may be a good candidate gene for breeding cold-tolerant crops.

## Materials and methods

### Plant materials and growth conditions

One-year-old grapevines (*Vitis amurensis* Rupr. var. ‘Zuoshan 1’) derived from the vineyard located in Gansu Agricultural University (Lanzhou, Gansu, China), were used to extract RNA. Tobacco (*Nicotiana benthamiana*), tomato (*Solanum lycopersicum* cv. ‘Micro-Tom’) seeds were purchased from Nanjing Fengshuo Horticulture Ltd., Company (Nanjing, Jiangsu, China). Tomato seeds were soaked using sterilized distilled water for 3 h, surface sterilized in 75% (v/v) ethanol for 30 s, and then treated with 2% (v/v) NaClO for 10 min, followed by four washes with sterilized distilled water. Seeds were planted on 1/2 Murashige and Skoog (MS) medium supplemented with 30 g/L sucrose and 6.5 g/L agar, pH was 5.8–6.0. Tomatoes with two cotyledons expansion about six days were used for genetic transformation. Transgenic tomato seeds (Transgenic generation 3, T3) and WT seeds were soaked in a conical flask with warm water for 30 min, and then placed on a shaking table until most seeds showed white sprouts. Seeds with white sprouts and tobacco seeds were planted in plastic plots with seedling substrate containing a mixture of soil and vermiculite (3:1), and cultured in growth chambers at 28 °C, 8 h, the light at 135 μmol·m^−2^·s^−1^/20 °C, 16 h, dark growth condition. The six-week-old T3 transgenic and WT tomato seedlings were used for the following cold assay. Four-weeks old tobacco seedlings were used for transient transformation.

### sDetailed information and evolutionary analysis of *VaPYL9*

The physicochemical property was analyzed by ProtParam in EXPASY (http://web.expasy.org/protparam/) including theoretical isoelectric point (pI), molecular weight, hydrophilicity, instability index, and grand average of hydropathicity (GRAVY). The protein is stable when the instability index is less than 40. Protein belongs to a neutral amino acid when GRAVY is in 0.5 ~ -0.5 (GRAVY < 0, strong hydrophilicity; GRAVY > 0, hydrophobicity). WoLF PSORT (https://wolfpsort.hgc.jp/) was employed to predict the subcellular localization of the *VaPYL9*. The protein sequences of PYL genes were downloaded from Phytozomev12.1: Home (https://phytozome.jgi.doe.gov/pz/portal.html), including 6 grape genes (*Vitis vinifera* L.), 16 *Arabidopsis* genes (*Arabidopsis thaliana*), and 16 tomato genes (*Solanum Lycopersicum*). Using these protein sequences, phylogenetic tree was constructed in MEGAX (Pennsylvania State University, State College, PA, USA) with the Neighbor-joining (NJ) algorithm setting 1000 bootstrap replicates [70]. Jalview was employed to perform multiple sequence alignment, and the muscle method with default parameter was adopted [71].

### Transient expression in tobacco

Four-weeks-old tobacco leaves were used to perform transient transformation for observing *VaPYL9* expression location in plant cell. The first strand cDNA of grape phloem was employed as a template to clone *VaPYL9* gene. The purified cDNA fragment was combined with *pART-CAM-EGFP* vector to form a fusion protein vector. The fusion plasmid with *pART-CAM-VaPYL9-EGFP* was introduced into *Agrobacterium tumefaciens* strain GV3101. *pART-CAM-VaPYL9-EGFP Agrobacterium tumefaciens* liquid was injected into tobacco leaves back, and then was incubated in the dark at 26 °C for 12 h. After dark incubation, injected tobaccos were placed in the light at 26 °C for 24 h. At last, green fluorescence was motivated in tobaccos and tobaccos leaves were snapped by confocal microscopy using laser scanning confocal microscope (Olympus FV1000 viewer) [72].

### Y2H protein binding assay

The CDS sequence of *VaPYL9* was cloned into pGBKT7 and fused to the DNA-binding domain to generate a bait construct. Primer sequences used in these assays are listed in Supplementary Table S1. The recombinant plasmid was transformed into Y2H Gold yeast strain, which was cultured on double dropout (DDO) medium (SD/ − Leu/ − Trp) for 3 days at 30 ℃ to detect self-activation and toxicity. PCR was used to verify the presence of *VaPYL9* in the Y2H Gold strain. The Mating hybridization method was adopted to screen potential interaction proteins from the AD library related to cold treatment which was constructed and preserved in Physiology and Biotechnology Laboratory (Gansu Agricultural University, China). Screened potential protein sequences were cloned into pGADT7 and fused to activation domain sequences to generate prey construct. Self-activation and toxicity detection were detected using the same method. The two constructs recombinant plasmids were co-transformed into Y2H Gold strain yeast cells and then cultured on a double dropout (DDO) medium (SD/ − Leu/ − Trp) at 30 ℃ for 3 days. After Co-transformation, the colonies were transferred to a quadruple dropout (QDO) medium (SD/ − Leu/ − Trp/ − Ade/ − His) with 10 mM 5-Bromo-4-chloro-3-indolyl-α-D-pyrangalactoside (X-α-Gal) to examine potential physical binding and interaction. Combinations of pGADT7-T with pGBKT7-53 and pGBKT7-Lam were served as positive and negative controls, respectively. All plates were incubated at 30 °C for 4 days and then photographed.

### Cloning of *VaPYL9* and obtaining of transgenic tomatoes

Based on our project's previous transcriptome sequencing on *Amur* grape phloem under cold stress, *VaPYL9* was screened and identified as a key gene from grape in cold stress. To further verify *VaPYL9* gene function, the full-length *VaPYL9* cDNA was cloned using grape phloem cDNA as a template into the overexpression vector pCAMBIA1301 digested by NcoI and BstEII enzymes, and *Escherichia coalition* (DH5α) carried recombinant plasmid was sequenced by Sangon Biotech (Shanghai, China) Co. Ltd. After successfully sequenced, the recombinant plasmid was transformed into *Agrobacterium tumefaciens* strain GV3101. *Agrobacterium tumefaciens* strain GV3101 with overexpression *pCAMBIA1301-VaPYL9* were transformed into WT tomato plants by the *Agrobacterium*-mediated leaf disk transformation method as described previously [73]. Gained sterile tomato cotyledons were cut as infection materials. Cut cotyledons were planted in MS (30 g/L sucrose + 6.5 g/L + 1 mg/L KT) to preincubate in dark condition for 2 days. Prepared infection bacteria solution using 1/2 MS suspension was used to immerse preincubated cotyledons for 5 min. Then infected cotyledons were coincubated for 3 days in dark condition. After cocultivation, they were transferred to generate callus and adventitious buds in MS (30 g/L sucrose + 6.5 g/L agar + 0.2 mg/L IAA + 2 mg/L ZT + 350 mg/L Timentin + 30 g/L hygromycin (Hyg). When the length of adventitious buds was about to 3 cm, cutting adventitious buds were transferred into MS (30 g/L sucrose + 6.5 g/L agar + 0.05 mg/L IAA + 350 mg/L Timentin + 30 g/L Hyg) to form roots. Transgenic plant lines were screened for 30 mg/L Hyg resistance and identified by the special primer according to instruction of FTL MIX (MF848) kit (Mei5 Biotechnology, Beijing, China Co., Ltd). Homozygous transgenic plants were selected in T1 generation derived from T0, and were stably maintained up to T3 generations. Ultimately, homozygous and stable T3 lines were gained.

### Cold assay

Three stable T3 transgenic lines were selected from previously gained tomatoes. By cooling plant culture chamber up to 4 °C step by step, some growth well and consistent six-week-old tomato plants including transgenic plants and WT plants were selected and placed in the culture chamber keeping 4 °C for 48 h. Tomato and grape leaves were collected in 0 h, 6 h, 12 h, 24 h, 48 h, quickly frozen in liquid nitrogen and stored at − 80 °C for qRT-PCR and determination of physiological indexes related to cold tolerance. Three biological replicates were applied in this experiment.

### Determination of H_2_O_2_, MDA, proline contents and antioxidant enzyme activities

H_2_O_2_ and malondialdehyde (MDA) content and catalase (CAT), superoxide dismutase (SOD), and peroxidase (POD) activities were performed as described in the instruction of the commercial ELISA kit purchased from Suzhou Comin Biotechnology Co.ltd (Suzhou, Jiangsu, China) and reaction mixtures were measured in Multimode Reader (Spark® Multifunctional microplate detector, TECAN, Pudong, Shanghai). The proline contents were determined as previously described [74].

### Determination of relative electrolyte leakage and content of IAA and ABA

The relative electrolyte leakage (REL) was measured using a model DDS307A device (Shanghai Leica Instrument) and the ratio of C1 (value of Leaf before boiling) to C2 (value of Leaf after boiling) was calculated as previously described [75]. 20 round leaves were gained using punches, and placed in glass test tubes with 15 mL ultrapure water. After 12 h, C1 was detected by a conductivity instrument. Then boiling for 15 min, C1 was detected. C1/C2% was viewed as relative electrical conductivity value. The content of IAA and ABA was determined by High Performance Liquid Chromatography (HPLC) mentioned in previous experiments [76]. Transgenic and WT Tomatoes leaves were ground and extracted using 5 mL 80% (V/V) chromatographic methanol at 4 ℃ for 12 h. During extraction, samples were constantly shaken to make hormone sufficiently dissolve. After centrifuged at 8000 rpm, 4 ℃,10 min, supernatants were transferred in new tubes. Repeat steps above once, supernatants were transferred in the same tube and constant volume was up to 10 mL. 2 mL supernatants were evaporated to dryness using a rotary evaporator. After evaporated to dryness, residue samples were thawed using 2 mL 50% (V/V) chromatographic methanol. HPLC condition was listed: Symmetry C18 chromatographic column (4.6 mm* 250 mm、5 μm), flowing phase (10% chromatographic methanol and 90%, 0.1% phosphoric acid), flow velocity (1 mL/min), sample volume (10 μL), determination waves length (254 nm), the temperature column (30 ℃). Hormones were detected in American Waters Acquity Arc high performance liquid chromatography.

### The DAB and NBT staining

3, 3, 9-Diaminobenzidine (DAB) and Nitrotetrazolium Blue Chloride (NBT) staining methods were performed as previously described [76, 77]. 10 mM 2-(N-Morpholino) ethanesulfonic acid (MES) was dissolved in distilled water and PH was 5.5. 1 mg/L DAB (Sigma-Aldrich) was dissolved in MES Buffer Solution and PH was 5.5. 0.5 mg/L NBT (Sigma-Aldrich) was dissolved in distilled water and PH was 5.8. Prepared DAB and NBT stain solution were stored at 4 ℃ and kept in a dark place. The destaining solution was ethanol: lactic acid: glycerin (3:1:1). Detached Leaves of tomatoes were immersed in culture dishes containing DAB and NBT staining solution for 12 h. The stained leaves were boiled with 95% ethanol for 10 min and leaves were decolorized with a destaining solution for 2 h. Decolorized leaves were placed in culture dishes with distilled water and photoed in EPSON Scan of root scanner (Regent company, Canada).

### RNA extraction and qRT-PCR

Total RNA was extracted from the leaves of grape and transgenic and WT tomato plants through RNAprep Pure Plant Kit (TIANGEN, Beijing, China). RNA was reversed as cDNA using the Prime Reverse Transcriptase Kit (Takara Bio, Shiga, Japan). Reversed cDNA was stored at − 20 °C and used as templates to amplify genes and perform qRT-PCR. All primers were designed on an online website and synthesized by Sangon Biotech (Shanghai) Co. Ltd (Supplementary Table S2). qRT-PCR was performed through the Light Cycler® 96 real-time PCR system (Roche, Switzerland). The *VvNAPDH* (GenBank accession no.CB973647) and *SlActin* (GenBank accession U60480) gene were used as internal controls. The 20 µL reaction solution contained 2 µL cDNA, 2 µL primers, 10 µL SYBR Green Master Mix Reagent (Takara Bio, Shiga, Japan), and 6 µL ddH_2_O. PCR amplification conditions were set as 50 cycles of 95 °C for 30 s, 95 °C for 10 s, 58 °C for 30 s, 72 °C for 20 s. The relative expressions of the genes were obtained from three biological replicates and calculated using the 2^−△△Ct^ method [78].

### RNA-seq library preparation and sequencing in tomatoes under cold stress

Based on comprehensive data analysis in tomatoes after cold stress, transgenic and WT tomato leaves at 4 ℃ for 48 h were screened for RNA-seq. RNA extraction was conducted as described above. RNA-seq was commissioned by Biomarker Technologies Co., Ltd. (Beijing, China). Three biological replicates of each sample were used for RNA-seq analysis. RNA quality and concentration were determined by agarose gel electrophoresis using NanoPhotometer spectrophotometer (Implant, Germany) and Agilent 2100 BioAnalyzer (Agilent Technologies, USA). RNA samples with inspection qualified were used to construct the library. Eukaryotic mRNA was enriched by magnetic beads with Oligo (dT). The mRNA was randomly interrupted by Fragmentation Buffer. Using mRNA as a template, the first cDNA strand was synthesized using random hexamers, and then the second cDNA strand was synthesized by adding buffer, dNTPs, RNase H and DNA polymerase I. The cDNA strand was purified using AMPure XP Beads. The purified double-stranded cDNA was then terminally-repaired, added Poly-A and linked to a sequenced adaptor. AMPure XP Beads were then made segment size selection. Finally, the cDNA library was obtained by PCR enrichment. The libraries were sequenced on an Illumina the Novaseq6000 platform. FPKM value of each gene was calculated to estimate gene expression levels [79]. Gene expression difference was estimated using the DESeq2 [80]. The differentially expressed genes (DEGs) were screened by setting false discovery rate (FDR) < 0.05 and |Log2 FC (fold change)|≥ 2 as thresholds screening measurement. Kyoto encyclopedia of genes and genomes (KEGG) enrichment analysis of DEGs was implemented by the cluster Profiler [81, 82].

### Statistical analysis

All data were determined in three independent biological replicates for each experiment. Data analyses were done by using one-way analysis of variance (ANOVA) in SPSS 22.0 (SPSS, Inc., Chicago, IL, USA). Duncan’s multiple range tests were employed to test significant differences (P < 0.01). Origin 9.0 was used to draw diagrams.

## Supplementary Information

**Additional file 1:** **Supplementary Table S1.** Detailed bioinformatic information of *VaPYL9* gene in grape.

**Addirional file 2:** **Supplementary Table S2.** All quantitative real-time primers for the expression level.

**Additional file 3:** **Supplementary Fig S1. **Multiply sequence alignment of PYL gene members among grape, tomato, *Arabidopsis thaliana*. Underline part was conserved domain (polyketide-cyc2) feature in PYL sequences. The length of *VaPYL9* domain sequence was 1-178.

**Additional file 4:** **Supplementary Fig S2. **The construction of *VaPYL9* transient expression vector. The presence of *VaPYL9* were verified including amplified cDNA (a), introduced into *Escherichia coalition* (b) and *Agrobacterium tumefaciens* strain GV3101 (c) by 1 % agarose gel eletrophoresis, respectively.

**Additional file 5:** **Supplementary Fig S3. **The construct of *VaPYL9 *overexpression vector. The presence of *VaPYL9 *were verified including amplified cDNA (a), introduced into *Escherichia coalition* (b) and *Agrobacterium tumefaciens strain* GV3101 (c) by 1 % agarose gel eletrophoresis, respectively.

**Additional file 6:** **Supplementary Fig S4. **The identification of transgenic tomatoes. Through *Agrobacterium*-mediated leaf disk transformation method, transgenic tomatoes were gained. Through screening, stable and homozygous lines (#2, #6, #9) were identified and named OE1, OE2, OE3 by 1 % agarose gel electrophoresis. These stable lines were used for the following experiment.

**Additional file 7:** **Supplementary Fig S5. **The construction pGBKT7-*VaPYL9* and pGADT7-*VaPCMT. *The presence of *VaPYL9* (a) and *VaPCMT *(b) were verified including amplified cDNA, introduced into Y2H gold by 1 % agarose gel electrophoresis, respectively.

**Additional file 8:** **Supplementary Fig S6.** Self-activation toxicity test of bait protein *VaPYL9* and prey protein *VaPCMT*. Dental plaque didn’t show blue. They were verified that there were no self-activation toxicity.

**Additional file 9:** **Supplementary Fig S7. **Co-transformation of *VaPYL9* and *VaPCMT. VaPYL9* and *VaPCMT* were together introduced into Y2H gold and its presence was identified in Y2H gold by 1 % agarose gel electrophoresis.

**Additional file 10:** **Supplementary Excel file. **Annotation information of DEGs in heatmap.

## Data Availability

All generated data in this study are included in supplementary information files. RNA-seq raw data have been deposited in NCBI (https://submit.ncbi.nlm.nih.gov/subs/sra/) under BioProject PRJNA830676.

## Abbreviations

ABA: Abscisic acid
PYR1/PYL/RCAR: Pyrabactin Resistance1/PYR1-Like /Regulatory Components of ABA receptors
Y2H: Yeast two-hybrid
WT: Wild-type
qRT-PCR: Quantitative real-time PCR
RNA-seq: Transcriptome sequencing
START: STAR-RELATED LIPID-TRANSFER
PP2C: Type 2C protein phosphates
SnRK2: SNF1-related protein kinase 2
MS: Murashige and Skoog
T3: Transgenic generation 3
PI: Isoelectric point
GRAVY: Grand average of hydropathicity
NJ: Neighbor-joining
DDO: Double dropout
QDO: Quadruple dropout
X-α-Gal: 5-Bromo-4-chloro-3-indolyl-α-D-pyrangalactoside
Hyg: Hygromycin
MDA: Malondialdehyde
CAT: Catalase
SOD: Superoxide dismutase
POD: Peroxidase
REL: Relative electrolyte leakage
HPLC: High Performance Liquid Chromatography
DAB: 3, 3, 9-Diaminobenzidine
NBT: Nitrotetrazolium Blue Chloride
MES: 2-(N-Morpholino) ethanesulfonic acid
DEGs: Differentially expressed genes
FDR: False discovery rate
FC: Foldchange
GO: Gene Ontology
KEGG: Kyoto encyclopedia of genes and genomes
PCMT: Protein-L-isoaspartate O-methyltransferase 1
