# Supplementary material for: Overexpression VaPYL9 improves cold tolerance in tomato by regulating key genes in hormone signaling and antioxidant enzyme
Source: BMC Plant Biol. 2022 Jul 15;22:344. doi: 10.1186/s12870-022-03704-8 (PMC9284830; doi:10.1186/s12870-022-03704-8)
Supplement: Supplementary file 1 — Additional file 1: Supplementary Table S1. Detailed bioinformatic information of VaPYL9 gene in grape. [file 12870_2022_3704_MOESM1_ESM.docx]

Additional file 1

**Table S1** Detailed bioinformatic information of *VaPYL9* gene in grape

| Gene name | Gene ID | [Gene](javascript:;) location | MW（ KD） | PI | Formulation | II | AI | GRAVY |
| --- | --- | --- | --- | --- | --- | --- | --- | --- |
|  |  |  |  |  |  |  |  |  |
| *VaPYL9* | GSVIVG01027078001 | chr15:18133348..18136210 forward | 20045.03 | 6.38 | C_875_H_1414_N_258_O_261_S_10_ | 37.65 | 95.67 | -0.193 |
| **Subcellular location prediction** | | | | | | | | |
| cytoskeleton | cytoplasm | nucleus | chloroplast | [extracellular](C:/Program%20Files%20(x86)/Youdao/Dict/8.10.3.0/resultui/html/index.html#/javascript:;) | mitochondrion | plasma | golgi bodies | vacuole |
| - | 10 | 1 | - | 2 | 1 | - | - | - |

Note：

MW: Molecular weight

PI: Theoretical isoelectric point

II: The instability index

AI: Aliphatic index

GRAVY: Grand average of hydropathicity
