# Supplementary material for: Overexpression VaPYL9 improves cold tolerance in tomato by regulating key genes in hormone signaling and antioxidant enzyme
Source: BMC Plant Biol. 2022 Jul 15;22:344. doi: 10.1186/s12870-022-03704-8 (PMC9284830; doi:10.1186/s12870-022-03704-8)
Supplement: Supplementary file 2 — Addirional file 2: Supplementary Table S2. All quantitative real-time primers for the expression level. [file 12870_2022_3704_MOESM2_ESM.docx]

Additional file 2

**Table S2** All quantitative real-time primers for the expression level

| gene | Gene ID | Forward primer for qRT-PCR(5'-3') | Reverse primer for qRT-PCR(5'-3') |
| --- | --- | --- | --- |
| *VaPYL9-OE* | GSVIVG01027078001 | GACACccatggtaGCTTTGGGGGGAATAGACGGCTG | GACACggtcaccTCAGACTGCATTGGCAGGTTCAAC |
| *VaPCMT-EGFP* | VIT_00007595001 | GGAGAGGACACGCTCGAGatgGCTTTGGGGGGAATAG | CCCTTGCTCACCATGAATTCGACTGCATTGGCAGGTTCA |
| *VaPYL9-BD* | GSVIVG01027078001 | CGGAATTCCCATGGTAGCTTTGGGGGGAA | TGGCTGCAGGGTCACCTCAGACTGCATTGGC |
| *VaPCMT-AD* | VIT_00007595001 | gtgggcatcgatacgggatccatATGCTATCTCCTCTTGTGTTAGCTTACA | cagctcgagctcgatggatccTCAATAATCGCGCAACTGAGC |
| *SlABI5* | XM_010327616.3 | CGGTAATGGAGGAGACAGCAATGG | CTAACCACACCAGCCTTCACCAAG |
| *SlNCED3* | Solyc07g056570.1 | CCGCAGTGGAATCTCTTCGTTCAG | TTCAAGACCAAGCGACCAACAGG |
| *SlCAT* | NM_001247257 | AAGTTCGCCATGCTGAGGTGTATC | TTCTTGTCTGTCGGGTGTGAATGAG |
| *SlPOD* | NM_001302921.2 | GAGAGGTCTGTTCCAATCCGATGC | ATTCGTTGAGTGGTCCATCTACAAGC |
| *SlSOD* | NM_001311084.1 | CTGTTGTTGTTCATGCTGATCCTGATG | GCCAATAATACCACAAGCAATCCTTCC |
| *SlActin* | GenBank accession U60480 | GGCAGACGGAGAGGATATTCA | TGACCCATACCCACCATCAC |
| *VvGAPDH* | GenBank accession no.CB973647 | TTCTCGTTGAGGGCTATTCCA | CCACAGACTTCATCGGTGACA |
| *GsSRK* | Solyc07g055630.4 | CCTGAATGGTTGAGGTCGGTGATC | TCGCCCACAAAGAACTTCCAAGAG |
| *SlPYL4* | Solyc03g095780.3 | AAACGGAACAGTGGTGGTGGAATC | TCAGCGATTTGCAGTTGCGATTTG |
| *SlPR5* | Solyc04g007380.3 | GCATCCAAACCGATCCGTCCAG | GTGGTAGTAGGCATGTCACTGTCTG |
| *SlWRKY* | Solyc03g116890.3 | TATGCTGCTGCGGTAATGCCAAG | TTACGCTTCTGACCACTTGCACTAC |
| *SlPYR/PYL* | Solyc09g090990.2 | ACAACCACAATTTCCCCAACAAGAC | CCAACACCACCATCTCCCTCAATAG |
| *SlMYC2* | Solyc09g065100.3 | TTCCAAGTTTGCCTCTTCTGCTACC | GCGACGACAGTGGGAGAGATTTG |
| *SlMAPK17/18* | Solyc02g090970.1 | TTGGGTTAGAGGTGAAACAGTTGGC | CTGAGCAGGAAGCGGAACAAGAC |

**Sequences were used in this experiment**

**>*VaPYL9***

ATGGCTTTGGGGGGAATAGACGGCTGTGATGCGTGCAACGCCATGATGGAGGCGCAGGTCATTTGTAGACATCATGCGCACGAGCCCCGGGAGAACCAGTGCAGTTCAGTTCTTGTTAGGCACGTCAAAGCTCCTGCTAATCTGGTTTGGTCACTGGTAAGGAGATTTGATCAACCCCAGAAGTACAAGCCCTTTGTTAGTAGGTGTGTGGTGCAAGGGGACCTCAGGATTGGGAGTGTCCGGGAAGTGAACGTCAAGACCGGTCTTCCGGCAACAACCAGCACGGAAAGGCTAGAGCTGTTTGATGACGATGAACACGTACTTGGCATCAAAATCCTCGATGGGGATCACAGACTAAGGAACTACTCCTCTGTTATTACTGTTCATCCAGAAATAATTGATGGGAGACCAGGGACACTGGTGATTGAGTCCTTTGTGGTGGACGTGCCTGAGGGGAACACCAAGGATGACACATGCTACTTTGTTCGGGCCCTCATCAACTGCAACCTCAAATGTCTGGCTGAAGTCTCAGAGAGGATGGCCATGCTGGGCCGAGTTGAACCTGCCAATGCAGTCTGA

***>PCMT***

ATGCTATCTCCTCTTGTGTTAGCTTACACTTGCCGCTATTGTGCGCCGCTTAACCACCTCTTAACCTTCACCCTCCACCAGCATCACCACCACCGGCACTCCCTCGCCATCTCTGCGCCCTCGACCACTCTATCTTGTCTGCATACCCCAAATCCTAGGTTCCTAACGGGGAACTCTCTCGTTTTCAAGATGGAGCGATTCTTTTCTGGAAGTGGGATCAATAAGAATAAAGCAATGGTGGAGCACTTGCAGCGCTATGGAGTGATTAATTCGAAAAAGGTAGCTCAAGTAATGGAGACTGTTGACAGAGCATTGTTTGTGCCTGATGGGAACCCACCTTATGTTGACAGCCCCATGCAGATAGGTTACAATGCCACTATTTCAGCACCTCATATGCATGCCACATGCCTCGAGCTGTTGCAGGAAAATTTGCAGCCTGGAATGCATGCTCTAGATGTTGGTTCTGGAACGGGGTATTTGAAGCCTGCTTTGCACTGATGGTTGGACCACAAGGTTGTGCAGTTGGAGTAGAACACATACCTGAGTTGGTTGCTTCTTCAATCAAGAATATCGAAAAGAGTGCAGCGGCTCCGTTATTAAAAGAAGGTTCTCTCAAACTGCATGCCGGTGATGGAAGGCTAGGTTGGCCTGAGTGTGCACCATATGATGCAATTCACGTCGGAGCCGCAGCACCTGAGATACCACAGCCACTTATTGATCAGTTGAAGCCCGGTGGTAGAATGGTCATTCCTGTTGGGAACATATTCCAGGATTTGAAGGTAGTGGACAAGAACCTGGACGGCTCCATCAGTATCCGCAGCGAGACTTCTGTCCGCTATGTTCCACTGACAAGTCGGGAGGCTCAGTTGCGCGATTATTGA

***>SlABI5***

ATGGGAGTACCAGAATCAGAGATGGTGTCTCAAAGTGAGGTTCAATCACCATTGCAACAAGACCAAAACCAGCACAAGAACAACCCATTCCCGTCACTCGGTCGACAAGCGTCGATTTACTCGCTCACGCTTGACGAATTCCAACACACTGTTTGCGAGAGTGGGAAGAATTTTGGGTCGATGAACATGGATGAATTTCTTAACAGCATTTGGACTGCTGAAGAAAATCAAGCCCACGCGCACGCTCAGCCTCACTGCCAGGCTGCAAGTACTGGGGAAGCAACTAGCGCCCCACGTTTTGCGTTAGGACAGGGAAATGTTTCGTTGGAGAAAGCTATTGTCGAGCAGCCAAGCTTGCCAAGACAGGGATCGCTTACGCTTCCTGCACCGTTGTGTAGTAAAACTGTGGATGAAGTTTGGTCAGAAATCCATAAGACCCAGCAAGAGCAGCAACAGAACAACGGGTGCAACATACAGAACACCGGTAACGGAAGTTCCACTCAACGACAGGCTACGTTCGGTGAAATGACGCTCGAAGATTTCTTGGTTAAAGCAGGGGTCGTACGCGAACAGGGCAATTCAGCTCCCGCACCTCCTCAGCAGCAATCATATATGATGTATCCAAACAGTGCAAATCCCACTATGGCCGCCATGGCTCGGCCTGTTATCGGCCTCGGTGGAGTCACAGGCGGTGTTGGCGTCGGTGTCTCCATTCCCGGTTATCCGCCACTTCCACAAACCGGGGTGGTCGAGGCACCTGTGTACCCTATGAGCATGAAAAGAGGCAGTGGATTCCCACAACAGTCAACCCCCGTCTACGGTGGTAGAATGGGAAACGGTAGCGGGGTTGGCTACGGGCAAGTAGTGCAAGGCGTAGCCGGAATGGGGTCGCCACTAAGTCCCGTGTCGTCGGATGCACTATGCGTAAATCAAATCGATAGCGGGGGCCAATACGGGTTGGAAATAGGAATGAGAGGCGGGCGAAAACGTGTACTAGACGGTCCAGTAGAGAAAGTTGTTGAAAGGAGGCAAAGGAGGATGATCAAGAACAGAGAATCCGCAGCAAGATCACGAGCAAGGAAACAGGCTTATACTGTTGAACTTGAGGCAGAATTGAATCAGCTAAAAGAAGAAAATGCACATCTAAAACAGGCCCTGGCGGAGCTCGAGAGGAAAAGGAAACAACAGTACTTTGATGAAGCGAAAATGAAAGCTCAAACGAAGGCGCAAAAGGCGAATGGCAAATTAAGAGGGATGAGA

AGGAGCTTGAGTTGCCCTTGA

***>SlNCED3***

ATGGCAACTACTACTTCACATGCCACAAATACATGGATTAAGACTAAGTTGTCAATGCCATCATCAAAGGAGTTTGGTTTTGCATCAAACTCTATTTCTCTACTCAAAAATCAACATAATAGGCAAAGTCTCAACATTAATTCCTCTCTTCAAGCTCCACCTATACTTCATTTTCCTAAACAATCTTCAAATTATCAAACACCAAAGAATAATACAATTTCACACCCAAAACAAGAAAACAACAACTCCTCTTCTTCTTCAACTTCCAAGTGGAATTTAGTGCAGAAAGCAGCAGCAATGGCTTTAGATGCTGTAGAAAGTGCTTTAACTAAACATGAACTTGAACACCCTTTGCCGAAAACAGCCGACCCACGAGTCCAGATTTCTGGGAATTTTGCTCCGGTACCGGAAAATCCAGTCTGTCAATCTCTTCCGGTCACCGGAAAAATACCCAAATGTGTTCAAGGCGTTTACGTTCGAAACGGAGCTAACCCTCTTTTTGAACCAACCGCCGGACACCATTTCTTCGACGGCGACGGTATGGTTCACGCCGTTCAATTCAAAAATGGGTCGGCTAGTTACGCTTGCCGTTTCACTGAAACAGAGAGGCTTGTTCAAGAAAAAGCTTTGGGTCGCCCTGTTTTCCCTAAAGCCATTGGTGAATTACATGGTCACTCTGGAATTGCAAGGCTTATGCTGTTTTACGCTCGTGGGCTCTTCGGACTTGTTGATCACAGTAAAGGAACTGGTGTTGCAAACGCCGGTTTAGTCTATTTCAATAACCGATTACTTGCTATGTCTGAAGATGATTTGCCTTACCATGTAAAGGTAACACCCACCGGCGATCTTAAAACAGAGGGTCGATTCGATTTCGACGGCCAGCTAAAATCCACCATGATAGCTCACCCAAAGCTCGACCCAGTTTCCGGTGAGCTATTTGCTCTTAGCTACGATGTGATTCAGAAGCCATACCTCAAGTACTTCAGATTTTCAAAAAATGGGGAAAAATCAAATGATGTTGAAATTCCAGTTGAAGACCCAACAATGATGCATGATTTCGCAATTACTGAGAACTTCGTCGTCATTCCTGATCAACAAGTCGTTTTCAAGATGTCTGAAATGATCCGTGGAGGTTCACCGGTGGTTTACGACAAGAACAAAGTTTCCCGATTTGGTATTCTGGATAAGTACGCGAAAGATGGGTCTGATTTGAAATGGGTTGAAGTACCTGATTGTTTCTGTTTCCACCTCTGGAATGCTTGGGAAGAAGCAGAAACAGATGAAATCGTTGTAATTGGTTCATGTATGACACCACCAGACTCCATTTTCAATGAATGTGATGAAGGGCTAAAGAGTGTTTTATCCGAAATCCGTCTCAATTTGAAAACAGGGAAATCAACAAGAAAATCCATAATCGAAAACCCGGATGAACAAGTGAATTTAGAAGCTGGAATGGTGAACCGAAACAAACTCGGAAGGAAAACAGAGTATGCTTATTTGGCTATCGCTGAACCATGGCCAAAAGTTTCTGGTTTTGCAAAAGTAAACCTGTTCACCGGTGAAGTTGAGAAATTCATTTATGGTGACAACAAATATGGTGGGGAACCTCTTTTTTTACCAAGAGACCCCAACAGCAAGGAAGAAGACGATGGTTATATTTTAGCTTTCGTTCACGATGAGAAAGAATGGAAATCAGAACTGCAAATTGTTAACGCAATGAGTTTGAAGTTGGAGGCAACTGTGAAGCTTCCATCAAGAGTTCCTTATGGATTTCATGGAACATTCATAAACGCCAATGATTTGGCAAATCAGGCATGA

***>SlCAT***

ATGGATCCTTACAAGTACCGTCCGTCAAGTGCTTTCAATTCACCTTTCTGTACCACTAATTCTGGTGCTCCTGTTTTTAATAACAATTCATCTCTTACGGTTGGTGCAAGAGGTCCTGTGTTGCTTGAGGATTACCATTTGGTGGAGAAACTTGCCAACTTTGACAGGGAACGTATTGCAGAACGTGTTGTTCATGCCCGAGGTGCTAGTGCCAAAGGGTTTTTTGAAGTTACTCATGACATTGCTCACCTTACCTGTGCTGATTTCCTTCGAGCTCCTGGTGTACAGACTCCAGTCATTGTGAGATTCTCTACTGTTATTCATGAAAGGGGTAGTCCTGAAACTCTGAGGGATCCTCGTGGTTTTGCTGTCAAGTTTTACACCAGAGAGGGAAACTTTGATCTGGTAGGGAACAACTTCCCCGTCTTCTTCATCCGTGATGGAATGAAGTTCCCTGACATGGTCCATGCTCTGAAGCCAAATCCTAAGTCCCATATCCAGGAGAATTGGAGGGTCCTTGATTTTTTCTCTCATCATCCCGAAAGCCTGCACATGTTCACTTTCCTCTTCGACGATATTGGTATTCCACAAGATTACAGGCATATGGACGGGTCTGGTGTCCACACATTCACATTGATCAACAGGGCTGGAAAATCAACTTATGTGAAGTTCCACTGGAAGCCCACATGTGGTGTCAAGTCTTTGTTGGAAGAAGAGGCAATCCGAGTCGGAGGAGCAAATCACAGCCATGCTACTCAGGACCTCTATGACTCTATTGCAGCTGGAAATTATCCTGAATGGAAGCTCTTCATTCAGATTATGGATCCAGAACATGAAGACAAATTTGACTTTGATCCACTTGATGTGACAAAAACTTGGCCAGAGGACATCTTGCCTTTGCAGCCGGTGGGAAGATTAGTTCTGAACAAGAACATTGATAACTTCTTTAATGAGAATGAGCAGCTAGCTTTCTGCCCTTCTATTGTGGTTCCAGGTGTTTATTACTCAGATGATAAGATGCTTCAAACTCGTATTTTCTCCTACTCTGATACCCAGAGGTATCGACTTGGACCAAACTATTTGCAACTTCCTGCTAATGCTCCAAAGTGTGCTCATCACAACAATCACTATGATGGCTCTATGAATTTTATGCACAGGGATGAGGAGATCGACTACTTCCCTTCAAGGTATGATCAAGTTCGCCATGCTGAGGTGTATCCTATTCCTTCAACAGTTTGCAGTGGCAAACGCGAGAAGTGTATCATTCAAAAAGAGAACAATTTCAAGCAACCAGGAGAAAGGTACCGCTCATTCACACCCGACAGACAAGAACGCTTTATTCGTCGGTGGGTGGAGGCCTTGTCTGATCCTCGTATCACTTATGAAATACGCAGCATTTGGATCACATACTGGTCTCAGGCTGACAAGTCTTTGGGTCAAAAGCTTGCATCTAGGCTTAATGTGAGACCA AGCATATGA

***>SlPOD***

ATGGCTTCATTTAGCTATTTGATGAGTGTTTTGGTATTATGTGTAATCATAGGTTATACAAATGCTCAATTAGAGCTTAATTTCTATGCTAAAAGCTGTCCAAAAGCTGAGAAAATTATTAAAGATTTTGTTCAGCAACAAGTTCCTAAGGCTCCAAATACTGCAGCAGCCATACTCAGAATGCATTTCCATGATTGCTTTGTCAGGGGTTGTGATGGATCTGTACTTCTCAATTTCACTTCGACTAACGGAAATCAAACTGAAAAACTAGCTAATCCTAATTTGACATTGAGAGGTTTCTCATTCATTGATGCTGTTAAAAGATTAGTTGAAGCTGAATGTCCGGGAGTTGTTTCTTGTGCTGATATTGTCGCGTTGGTTGCTAGAGATGCAGTTGTGGCTACGGAGGGTCCTTTTTGGAATGTGCCAACTGGTAGAAGAGATGGAACGATATCAAATGTGTCAGAAGCCAATGGTGATATCCCAGCACCAACTAGTAACTTTACTAGACTGCAACAATCCTTCGCGAAGAAAGGTCTTGATCTGAATGACCTGGTCCTTCTATCAGGTGCCCATACTATTGGAGTGTCTCGTTGCTCATCATTTTCAGAGCGTCTATACAATTTCACCGGGGTTGTAGGTACACAAGATCCATCTCTAGACAGTGAATATGCGGATAATCTCAAGTCAAGAAAATGCAGATCAATCAATGACAATACTACTATAGTAGAAATGGATCCAGGTAGTTTCAAGACATTTGATCTCAGCTACTTCAAGCTTTTGCTCAAAAGGAGAGGTCTGTTCCAATCCGATGCAGCATTGACAACACGTACCTCAACGAAATCGTTTATCGAGCAGCTTGTAGATGGACCACTCAACGAATTTTTCGATGAATTTGCTAAATCGATGGAGAAAATGGGAAGAGTTGAAGTTAAGACAGGGAGTGCTGGTGAAATCAGGAAGCATTGTGCATTTGTGAATAGTTAA

***>SlSOD***

ATGGTGAAGGCCGTCGCCGTCCTTAACAGCAGTGAAGGTGTTAGTGGCACCATCCTCTTCACTCAAGATGGAGATGCTCCAACCACAGTTAATGGAAATATTTCTGGCCTAAAACCTGGACTTCATGGCTTCCATGTCCATGCCCTTGGTGATACCACAAATGGCTGTATGTCAACAGGACCACATTACAATCCTGCTGGTAAGGAGCATGGTGCTCCTGAAGATGAGGTGCGTCATGCTGGTGATCTTGGTAACATCACAGTTGGAGAAGATGGTACTGCATCTTTTACTATTACCGACAAGCAGATTCCTCTCACTGGTCCACAGTCCATCATTGGAAGAGCTGTTGTTGTTCATGCTGATCCTGATGATCTTGGAAAGGGAGGACATGAGCTCAGTAAAAGCACCGGAAATGCTGGCGGAAGGATTGCTTGTGGTATTATTGGCCTCCAGGGTTAA

***>SlActin***

GCTGGGTTCGCAGGAGATGATGCTCCACGAGCTGTATTTCCTAGTATTGTTGGCCGCCCCCGCCATACTGGTGTGATGGTGGGTATGGGTCAAAAAGACGCCTATGTGGGAGATGAAGCTCAATCGAAGAGAGGTATTTTAACTCTTAAATACCCAATTGAGCACGGAATTGTCAGCAATTGGGATGATATGGAGAAGATATGGCATCATACTTTCTACAATGAACTTCGTGTTGCCCCTGAGGAGCATCCTGTCCTCCTAACTGAAGCCCCTCTTAACCCAAAGGCTAATCGTGAAAAGATGACCCAGATTATGTTTGAGACTTTCAATACCCCAGCTATGTATGTTGCTATTCAGGCTGTACTCTCACTGTATGCCAGTGGTCGATCCACCGGTATTGTGTTGGACTCTGGTGATGGTCTCAGCCACACTGTCCCAATTTATGAAGGGTATGCCCTTCCACATGCCATTCTCCGTCTTGACTTGGCAGGACGTGACCTCACTGATAGTTTGATGAAGATCCTGACCGAGCGTGGTTACTCGTTCACCACCTCAGCTGAGCGAGAAATTGTCAGGGACGTGAAAGAAAAGCTCGCTTACATAGCTCTTGACTATGAACAGGAACTCGAGACCTCAAAGACCAGCTCTTCTGTTGAGAAGAGCTATGAGCTCCCAGATGGGCAGGTGATACCCATTGGGTCTGAGCGTTTCCGGTGTCCTGAGGTCCTTTTCCAACCTTCAATGATTGGAATGGAAGCTGCAGGAATCCACGAGACTACATACAACTCTATCATGAAATGTGACGTGGATATTAGGAAAGATCTTTATGGAAACATTGTGCTCAGTGGTGGTACTACCATGTTCCCAGGTATTGCTGATAGAATGAGCAAAGAAATTACTGCATTGGCTCCTAGCAGCATGAAGATTAAGGTGGTCGCTCCACCAGAGAGGAAATACAGTGTCTGGATTGGAGGCTCTATCTTGGCTTCCCTCAGCACCTTCCAGCAG

***>GAPDH***

ATGGCCAAGATCAAGATCGGAATCAATGGATTTGGAAGGATTGGTCGGTTGGTTGCCAGAGTTGCTTTGCAGAGAGATGATGTTGAACTTGTTGCTGTCAATGATCCTTTTATCAACACTGACTACATGACCTACATGTTTAAGTATGATTCTGTCCATGGCCAATGGAAGCATCATGATATTAAAGTGAAGGACTCCAAGACCCTTCTTTTTGGAGACAAGGCAGTTACTGTTTTTGGCGCGAAGAACCCTGAGGAGATCCCATGGGGTGAGGCTGGAGCCGAATATGTGGTCGAGTCCACTGGTGTTTTCACTGATAAGGACAAAGCTGCCCTGCATTTGAAGGGTGGTGCCAAGAAGGTCATCATTTCTGCTCCAAGCAGTAACGCTCCTATGTTTGTTATGGGTGTTAACGAGAAGGAATACAAGTCAAATATTGACATTGTTTCTAATGCTAGCTGCACGACCAACTGTCTTGCTCCATTGGCAAAGGTCATCCATGACAAATTTGGTATTGTTGAGGGTCTTATGACCACTGTCCATTCCATCACTGCTACACAAAAGACAGTTGATGGACCATCAATGAAGGATTGGAGAGGTGGCAGAGCTGCCTCTTTCAACATTATTCCCAGCAGCACTGGTGCTGCAAAGGCTGTTGGGAAGGTGCTACCAGCTCTGAATGGCAAATTGACTGGAATGGCTTTCCGTGTGCCCACGGCTGATGTCTCAGTGGTTGATCTCACTGTGAGGACGGAGAAGAAGGCCTCTTATGATGATATCAAGGCTGCCATCAAGGCGGAATCTGAGGGAAATTTGAAGGGAATCCTTGGTTACACTGAGGATGAAGTGGTATCAAGCGACTTTTTGGGAGACAGCAGGTCGAGTATTTTCGACGCCAAGGCTGGAATTGCTCTGAATGAAAACTTTATCAAGTTGGTCTCTTGGTATGATAATGAATGGGGTTACAGCTCGCGCGTGGTTGACTTGATCAGGCACATTG ACTCCACAAAATGA

**>*SlMAPK17/18***

ATGGATTGGGTTAGAGGTGAAACAGTTGGCCATGGAAGCTTTGGCAAAGTTAGTTTTGTGATTCCGAGAAACCAGAGTACTCTGTTTTCTCCATCAATGGTGGTTAAGTCTTGTTCCGCTTCCTGCTCAGCTACTTTGATGAACGAGAAGATAATCTTGGATGAACTTAAGGGGTGCCCACAAATAATCAATTGCGTTGGTGACAGCTACAGTTATGAAAATGGCGAAAAGCTATACAATGTCTTGTTGGAGTATGCTTGTGGGGGTGCTTTGTCGGATAAATTAAAGAATTCCGGTGATCAGAGGTTGCCGGAGCTTGAAGTCAGGGAGTACACAAAGGGGTTACTCAGAGGGATTCATTATATCCACAAGAATGGTTTTGTTCACTGCGACATAAAGCTTCCAAACATTCTTTTAGGCGAAAATGGTCAAGTAAAAATTGCTGATTTCGGATTGGCAAAGAGAGCGGAATCAAAGAGAGATGATAAATTGAGATGTGAATTGAGGGGTACTCCACTGTACATGTCACCGGAAATGGTGATCGGAGGCGAACAGAATACTCCGGCTGATATCTGGGCACTTGGCTGTGTGGTGGCGGAAATGGCAACTGGTAATCCAGTGTGGAGATGTTTAGATATAAGTAAATTGTTGATGACAATTGGATTGGGTGACCAATTACCTGAAATTCCTCAAAATTTATCGGAAGAAGGAAAAGATTTTCTTGAAAAATGCTTGATGAAGGACCCGAAAAAGAGATGGACAGCTGAGATGCTTCTAGAACATCCCTTTGTTGCTGATGAAGATGACACTGTTTTATTAAATTACGAAAGATGCAACAGTGGCAGCCCTTCAACATCTCCAAGATGCCCATTTGATTTCCCAGATTGGGTATCTAACAACTCTGCTGAATCCTCAGTAACATGTTCAATTACATCACTGCCCTCGCCGGCGTTTCAAGAATTGATGAACTGGAACGACGGGTCATGGTCTACATCGCCGACAGAGAGAATTCGAGAATTAGTGTGTGAACGTAAACCTGAATCTGAATGGTCTACAGCTGATGGCTGGGTCAGTGTTAGGTGA

**>*SlPYR/PYL***

ATGGGTGTGAATACCTATACTTATGAGTCAACAACCACAATTTCCCCAACAAGACTATTCAAAGCTTTGGTTCTTGATTTTGACAACCTTGTACCTAAATTGTTGTCACAACATGTTAAGAACAATGAGACTATTGAGGGAGATGGTGGTGTTGGAAGCATCAAGCAAATGAACTTTGTTGAAGGTGGTCCAATAAAGTACTTGAAACACAAGATTCATGTGATTGATGACAAGAACTTAGAAACAAAATATTCACTTATTGAAGGTGATATTCTTGGAGAAAAATTGGAATCAATTACTTATGATATCAAATTTGAAGCTAATGATAATGGAGGTTGTGTTTACAAGACAACAACTGAGTATCACACAAAGGGTGATCATGTTGTTAGTGAAGAAGAACACAATGTAGGCAGAGAGAGAATCATGAATATTTCCAAGGCTGTAGAAGCATACCTTCTCGCGAATCCTTCTGTCTACGCTTGA

**>*SlMYC2***

ATGGAGATTATACAGCCTAATAGCCTGCAGTTACAAAACATGTTGCAAAATTCCGTCCAGTCGGTTAAATGGACTTATAGTATTTTTTGGCAATTTTGTCCAAAACAAGGGGTGTTAGTGTGGAGAGATGGATATTATAATGGAGCTATAAAGACTAGAAAAACTGTGCAGCCAATGGAAGTTACTGCTGAAGAAGCTTCTCTTCATAGAAGCCAACAACTTAGAGAACTTTATGATTCACTTTCTGCCGGTGACTCAAATCCCCCGGCGAGAAGGCCGTCCGCAGCATTGTCGCCGGAGGATTTGACGGAATCTGAGTGGTTTTATCTCATGTGTGTTTCTTTCTCTTTTCCTCCACCTATTGGATTACCAGGCAAGGCTTATTCAAAGAAACATCACATATGGATAATGGGAGCAAATGATGTTGATAGCAAAGTCTTCTGTAGAGCTATTCTTGCTAAGACTGTAGTTTGTATTCCTCTCTTGGACGGTGTTGTGGAACTCGGAACTATAGAAAAGGTTCAAGAAGATATTGGATTTATACATCGCGTAAAAAGTTTCTTCAATGAGCCACAACAAGCTCAGCCACCAAAGCCAGCTTTATCTGAGCACTCCACTTCGGATCCCGCCGCCTTTTCGGAGCCACATTTTTACTTCAGCAATACTCCGTCATCTGCGGGTATTTGTCCAGCGGATCAAGACGGTAGAATTACTGGAGAAGAAGAAAATGAGGACGAGGACGAGGACGAGGCTGAGGATGACGAGGATGAAAATGATGAAGCTGAATTAGACTCGGATGGTATAGCAATTCAAAGTGGGGCTGGAGCGGCTAATCCTATGGCGGCTGAGGCTAGTGAGCTCATGCAGCTTGATATGTCTGAAGCTATACGGCTCGGCTCACCGGATGATGGCTCTAATAATATGGACACTGATTTATATTTGGATGGTATTAGCCAAGCTGGAAATACGGCTGACTCTTTCAAAGCTGAGACTGCAATTAGTTGGGCTAACTTCCAAGACCTTCAACATTTACCAGGTATACCTAGTTATGATGAATTATCACAAGAAGATACACATTATTCTCAAACAGTTTCAGCTGTACTTGAACACCTCTCGAACACAAGTTCCAAGTTTGCCTCTTCTGCTACCATAATGGGCTCTATTTCTCCTGATTCAGCCCAATCCGCTTTCACATTATGGCCCGTCACTTGTAGCCCAAATCTCTCCCACTGTCGTCGCCACGATATCGGCGATGGCAGTGGGACCACCTCTCAATGGCTGCTCAAAAGCATACTATTCACTGTCCCATTTCTCCACAGTACTAAAAAATTATCAGAAGCTCTGTCTCCAAAGTCACGAGACGCTGCTGCGGCCGACTCTTCGGCCGCAGCGTCTCGATTCCGCAAAGGATGTACGATAAATAGTTGTACACAACAAGAAGAGACGAGCGGGAACCATGTACTAGCGGAACGACGTCGTAGAGAGAAGCTGAATGAGCGTTTTATTATTTTAAGGTCCTTGGTACCCTTCGTTACGAAAATGGATAAAGCATCGATACTTGGCGATACAATAGAGTATGTGAAGCAGTTACGTAAGAAAGTTCAGGATCTCGAAGCGCGTGATCGCCACACGGAAATTACCAAAAAATCAGATGAAAAGAGTGGTTCACCAATAGTAAAGGCGTTTCCGGTGAAGGGTAAGAGGAGAATGAAGAGTACGGTGGAGGGAAGTATAGTCGGAGCACCGGCAAAGATGACGGGTTCTCCACCGATGGAAGAGGAGGTTTTGCAAGTGGAAGTCTCGATCATCGAAAACGATGCACTGGTGGAGCTCCGGTGTCCGTACAAAGAAGGGTTGTTGTTAGATGTAATGCAAGTTCTAAGGGAACTAAAGGTGGAAGTTGTAGCAATCCAATCATCTCTTAGTACTGGACTCTTATTGGCTGAGTTAAGAGCTAAGGTAAAAGAAAATATATATGGAAGGAAGGCAAGCATATTGGAAGTGAAAAAGTCAATAAATCAGATAATCCCTAGAGTTAATTAA

**>*SlWRKY***

ATGGAGTTCACAAGTTTAGTTGATACTTCTCTGGATTTGAACTTTAGACCTCTTCGAGTTTCCGATGAATTACCAAAACAGGAAGTTGAGAGTAATTTCATAGGACTTGGAAGAGATCTGGTACCTGTAAAAGATGAGGCAAGTAATTTAATAGAGGAACTAAATAGAGTAAATGCTGAAAATAAGAAATTGACGGAGATGTTAACAGTTATGTGCCAGAATTACAATTCATTGAGGAACCAATTGACGGAATATATGAGCAAGCAGAATAGTAGTACTAGTGGAGCTGATCAGGATCAGAACAGCGATGGATCGAAGAAAATTAAAATTGAAAACAACAACAATAATAATAATAATAATGAAATTGTGAAATCGTCAGTTCAAGTGTTGAATTCAGAGAGCAGCTCAAGTGATGAAGATTCATCTACAAAGAAACCAAGAGAAGAACACATTAAAACTAAGACTTCAAGAGTTTATATGAGAACTGAACCATCTGATACTTCTCTTATAGTGAAAGATGGATACCAGTGGAGGAAATATGGACAGAAAGTAACAAGAGACAATCCATCTCCAAGAGCTTATTTCAAATGCTCTTTTGCTCCTACCTGTCCCGTTAAGAAAAAGGTTCAAAGAAGCGTGGAAGACCAATCGATTCTAGTAGCAACCTATGAAGGAGAACACAACCATTCTAAAGTGGATACCGCAGGCCCTGTTACAACAACTTCCCCGTCTAGCCGATTTAACCCGAAAAATAATACTTATGCTGCTGC

GGTAATGCCAAGACAAACCTTAACTCTTGATTTGGCAGAACCAAAAACATTACAAAATGATATCAAAAAAGTTCATAGCATTACAAGTACAAGTAGTGCAAGTGGTCAGAAGCGTAAATCACCAGGAACTGATCAACAACAGCAAAATAGACCAGAGTTTCAACATTTCTTGATAGAACAAATGGCTTCATCATTGACTAAAGATCCAAGTTTTCAAGCAGCCTTAGCAGCCGCCATATCAGGAAAATTCTTGCAAAATAATAGTAACACTAAGGATAAATAA

**>*SlPYL4***

ATGCCTTGTTCAGTTCAGCTGCAGAGAATCAACCCCACAACCACCACCACTCTCGCCGGAAACTTCCACAAACCGCCTCAACCGACATGTATTATCCCGGTGCAGTTCTCCGTCCCTAATAATCACTTACATTACCACACCCACGCTGTAAGCCCCAACCAGTGCTGCTCTGCCGTGGTGCAGACGATCTCCGCCCCGATCCACGCCGTATGGTCACTAGTCCGCCGATTCGACAACCCGCAAGCGTATAAGCATTTTCTCAAAAGCTGCCACGTCATCGTCGGAGACGGGAACGTCGGCACATTAAGGGAGGTTCAGGTAATTTCCGGCCTCCCAGCCGCCTCTAGCACGGAGAGATTGGAAATCTTAGACGATAAGGAACACGTGATCAGTTTCAGCGTCGTCGGCGGAGATCACAGGCTGAACAATTACAGATCGGTGACGACGCTGCATCCGGCGGATGATGAAAACGGAACAGTGGTGGTGGAATCGTACGTAGTAGATATTCCAACAGGGAATACAAAGGAAGAAACGTGTGTATTCGTTGATACAATTGTACGTTGCAATTTGCAATCGCTAGCGCAAATCGCAACTGCAAATCGCTGA

**>*SlPR5***

ATGCTAGTTCTCCTATTCCTCTACTTAAGAAAAAAATGCTCCTTCATTTTCTGGAAGAGGAAAGGAGAGGATTATCGAAATGTAAAAGCCTTTTTGAAGAGCCGTGGATCACTCGCGCTAAGGAAGTACAGTTATTCTGAAGTTAAAAAGATGACCGAGTATTTCAAAAACAAACTTGGTCAAGGAGGCTATGGTTCTGTGTATAAAGGAAAGTTGCATACCGGGAGTCTCGTGGCAGTCAAGGTCTTGAAAGAATCGAAGGGCGGGGGAGAAGAGTTTATCAATGAGGTAGCAAGTATCAGTAGGACTTCTCATATTAATATTGTATCACTTGTGGGATTTTGTTTTGAAGGTCAACACAGAGCTCTCATCTATGATTTCATGCCTAATGGATCCCTTGAGAAGTTCATTTACGATGCAAAATGTGGTACGAATCATCAACTAGGATGGCATACATTGTACAATATTTCACTTGGCATTGCTAGAGGATTGGAGTATTTGCATCGCGGTTGCAATACTAGAATCCTGCATTTCGATATAAAGCCTCATAACATTCTTCTTGATGAAGACTTTTGTCCAAAAATATCCGATTTTGGCCTTGCAAAACTATGTAACAATAAGGAAAGCGTTGTGTCTTTATTGGGCGCGAGAGGGACTATTGGCTATATTGCACCAGAAATTGTGTGTAAAAACATTGGAGGAGTTTCTCACAAGTCAGATGTGTACAGCTATGGTGTGATGGTCCTCGAGATGGTTGGAGGAAGGAAAAATGTTGACGAAGTTATTGATTGTACTAGTGAAATATACTTCCCACACTGGATTTACAAACAGATTGAACAGAAGAAAGAGCTTGGATTAACTGGTATTGTGGAGGAAGAGGATAAGAAACTTGCAGAAAAGATGATACTGGTGAGCCTGTGGTGCATCCAAACCGATCCGTCCAGCAGGCCTTCTATCAGTATGGTCATAGAGATGTTACAAGGTGAACTCGAATCTCTGCAAATGCCTCCGAAGCCTTTCTTGTATTCTTCTTCAGTGTCAGACAGTGACATGCCTACTACCACCTACATGATATCAAGACTCGAAAGATCAAGTTTGATGATCTAG

**>*SlGsSRK***

TTGAATTCTACTACTGGACTTTCCAATTCTTGGATCAATATGCCATATGTTAGAGTTTACAATACCTCCGAGCCTTCTGAAATGACGCCTATTATTCGGAGCAGAAAGAATAGCCCTCAGTTTCTCTTTGCCTTCTATTGCCCCCTTATAACTAACAGTACCACAGAATGCCTTCTTGGTATCCTTTTATATTACAACAGATCCGACGGGGTTACGAGTACTCCCCAGTTAGTTTGGTCTGCTAACAAGAACCATCCAGTGAAAGTCAATGCAACCTTGGAACTAGGCCAAGATGGCAACTTGGTCTTGACAGACTCTGATGGCACTCTTGTTTGGTCCACTAATACTACCGGAAAATCTGTTTTTGGCTTAAACATGACAGAAATGGGAAATATTGTGCTCTTTAATAAGAGAAAGCAAGCAATTTGGCAGTCTTTTGATAATCCAACAGATTGTTTGCTTCCGGGACAGAAGTTGGTTTCGGGAAGGAGACTGATAGCAAGCAGTTCAGGATCCAATCAGTCCTTTTCTTTTACTGTTCTTGATGGAAGCTTGAGGACACTGTCTGTCAAGGCTGGGGATCTTCTGGATTTAGCACCAATCTTACCGGGAATCCTAACTCGATTCTCTTACAATGAGTTGAAAATAATTACACAAGATTTCAGCAGAAAGCTCGGGGAAGGAGGATTTGGCTCAGTATATGAAGGAACACTGAGTAATGGCAACAAAATAGCTGTGAAGCGTCTGAATGGTGTAGGTCAAGTAAAGGATTCATTCTTAACAGAGTACATGGTAAATGGATCACTGGATAGGTGGCTTTCTCATGAAAATCAAGAAAACGGGCTTACATGGCTTACGAGGCAAAAGATAATATCAGATATCGCTAAAGGATTAGCTTATCTTCATGATGAATGCAGTCAGAAGATAATTCATTTGGACATCAAGCCACACAACATCCTCTTGGATGAGAACTTCAATGCTAAGATATCTGATTTTGGGTTGTCGAAACTAATTGAGAAAGACAAAAGCAAAGTTGTAACTCGAATGAGAGGAACACCAGGGTATTTAGCTCCTGAATGGTTGAGGTCGGTGATCACTGAGAAAGTAGACGTATATGCCTTTGGAATTGTACTCTTGGAAGTTCTTTGTGGGCGAAAGAATTTGGATTGGTCACAGGCTGATGAAGACAATGTCCATTTGCTTAGTGTTTTTAGGAGAAAAGTAGAACAAGAGAAGCTCATCGATATGGTTGACAAGAACAACGAGGATATGCAGCTCCACAGGGAAGCAGTGACGGAAATGATG
