# Supplementary material for: Overexpression VaPYL9 improves cold tolerance in tomato by regulating key genes in hormone signaling and antioxidant enzyme
Source: BMC Plant Biol. 2022 Jul 15;22:344. doi: 10.1186/s12870-022-03704-8 (PMC9284830; doi:10.1186/s12870-022-03704-8)
Supplement: Supplementary file 3 — Additional file 3: Supplementary Fig S1. Multiply sequence alignment of PYL gene members among grape, tomato, Arabidopsis thaliana. Underline part was conserved domain (polyketide-cyc2) feature in PYL sequences. The length of VaPYL9 domain sequence was 1-178. [file 12870_2022_3704_MOESM3_ESM.docx]

Additional file 3

**Fig. S1** Multiply sequence alignment of PYL gene member among grape, tomato, *Arabidopsis thaliana*. Underline part was conserved domain (polyketide-cyc2) feature in PYL sequences. The length of *VaPYL9* domain sequence was 1-178.

**
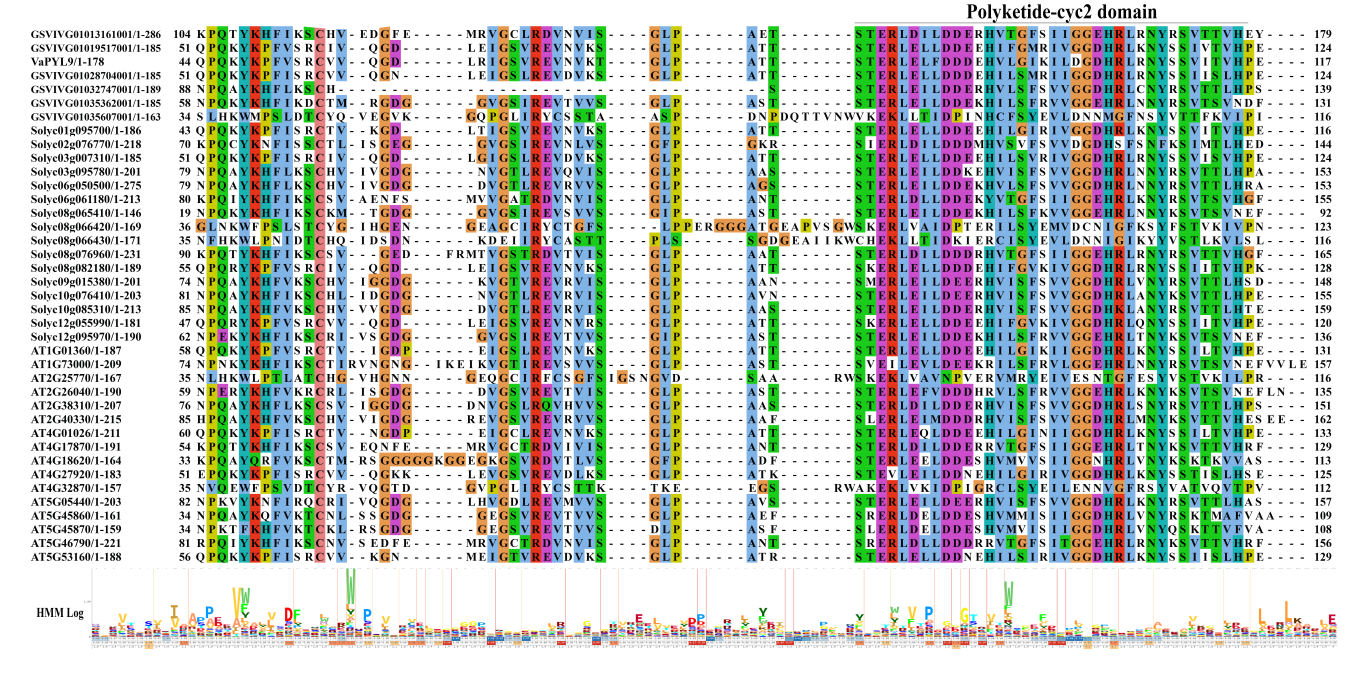
**
