# Supplementary material for: Overexpression VaPYL9 improves cold tolerance in tomato by regulating key genes in hormone signaling and antioxidant enzyme
Source: BMC Plant Biol. 2022 Jul 15;22:344. doi: 10.1186/s12870-022-03704-8 (PMC9284830; doi:10.1186/s12870-022-03704-8)

**Additional file 5**

**Fig. S3** The construct of *VaPYL9* overexpression vector. The presence of *VaPYL9* were verified including amplified cDNA (a), introduced into *Escherichia coalition* (b) and *Agrobacterium tumefaciens strain* GV3101 (c) by 1 % agarose gel eletrophoresis, respectively.


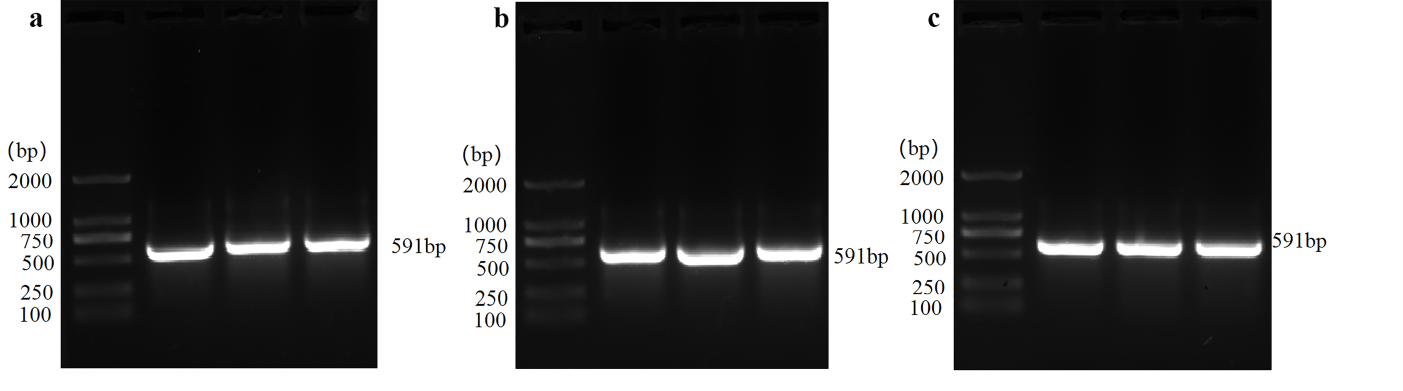

Supplement: Supplementary file 5 — Additional file 5: Supplementary Fig S3. The construct of VaPYL9 overexpression vector. The presence of VaPYL9 were verified including amplified cDNA (a), introduced into Escherichia coalition (b) and Agrobacterium tumefaciens strain GV3101 (c) by 1 % agarose gel eletrophoresis, respectively. [file 12870_2022_3704_MOESM5_ESM.docx]
