# Supplementary material for: Overexpression VaPYL9 improves cold tolerance in tomato by regulating key genes in hormone signaling and antioxidant enzyme
Source: BMC Plant Biol. 2022 Jul 15;22:344. doi: 10.1186/s12870-022-03704-8 (PMC9284830; doi:10.1186/s12870-022-03704-8)

**Additional file 6**

**Fig. S4** The identification of transgenic tomatoes. Through *Agrobacterium*-mediated leaf disk transformation method, transgenic tomatoes were gained. Through screening, stable and homozygous lines (#2, #6, #9) were identified and named OE1, OE2, OE3 by 1 % agarose gel electrophoresis. These stable lines were used for the following experiment.


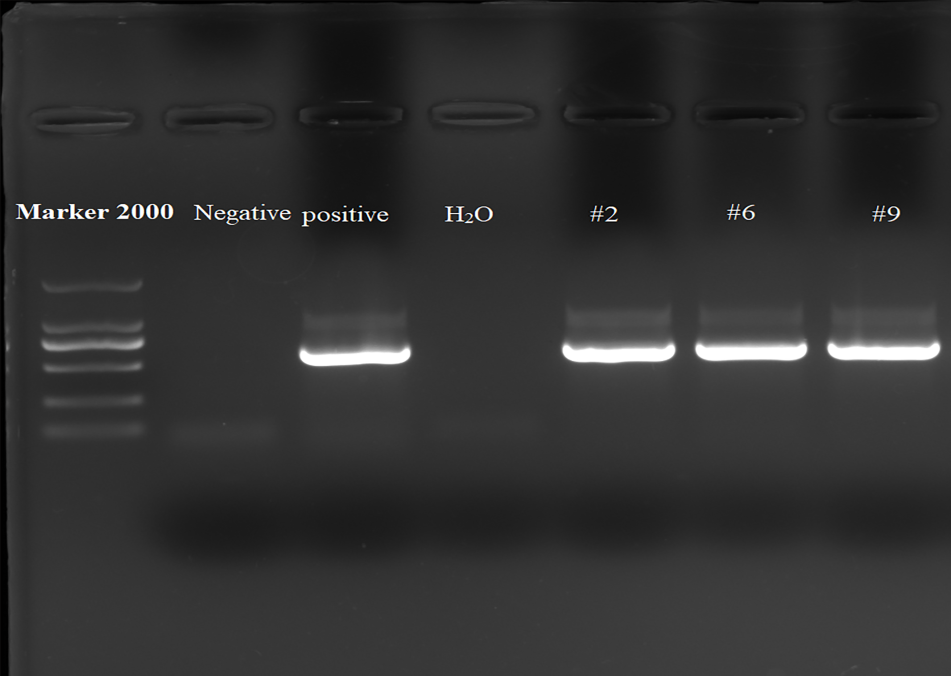

Supplement: Supplementary file 6 — Additional file 6: Supplementary Fig S4. The identification of transgenic tomatoes. Through Agrobacterium-mediated leaf disk transformation method, transgenic tomatoes were gained. Through screening, stable and homozygous lines (#2, #6, #9) were identified and named OE1, OE2, OE3 by 1 % agarose gel electrophoresis. These stable lines were used for the following experiment. [file 12870_2022_3704_MOESM6_ESM.docx]
