# Supplementary material for: Overexpression VaPYL9 improves cold tolerance in tomato by regulating key genes in hormone signaling and antioxidant enzyme
Source: BMC Plant Biol. 2022 Jul 15;22:344. doi: 10.1186/s12870-022-03704-8 (PMC9284830; doi:10.1186/s12870-022-03704-8)

**Additional file 7**

**Fig. S5** The construction pGBKT7-*VaPYL9* and pGADT7-*VaPCMT.* The presence of *VaPYL9* (a) and *VaPCMT* (b) were verified including amplified cDNA, introduced into Y2H gold by 1 % agarose gel electrophoresis, respectively.


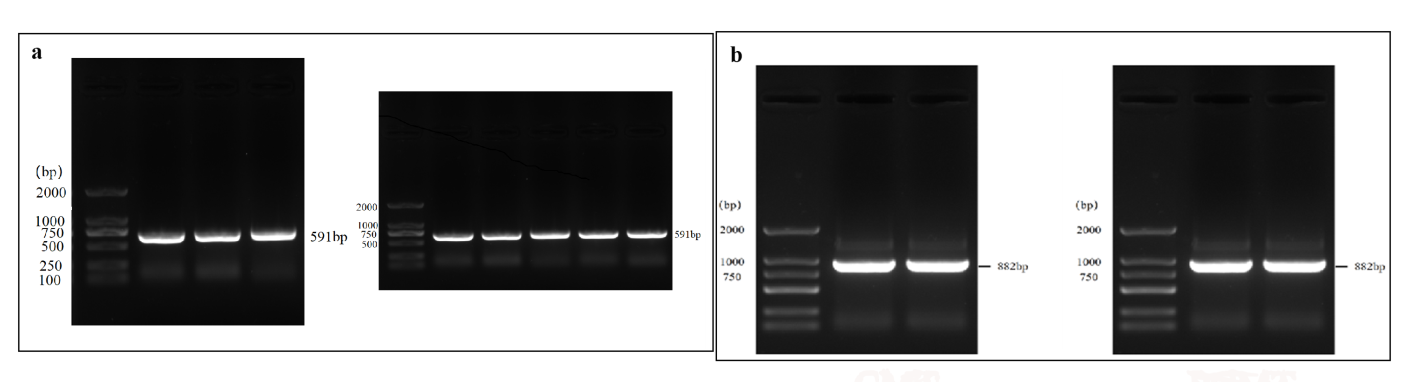

Supplement: Supplementary file 7 — Additional file 7: Supplementary Fig S5. The construction pGBKT7-VaPYL9 and pGADT7-VaPCMT. The presence of VaPYL9 (a) and VaPCMT (b) were verified including amplified cDNA, introduced into Y2H gold by 1 % agarose gel electrophoresis, respectively. [file 12870_2022_3704_MOESM7_ESM.docx]
