# Supplementary material for: Overexpression VaPYL9 improves cold tolerance in tomato by regulating key genes in hormone signaling and antioxidant enzyme
Source: BMC Plant Biol. 2022 Jul 15;22:344. doi: 10.1186/s12870-022-03704-8 (PMC9284830; doi:10.1186/s12870-022-03704-8)

**Additional file 8**

**Fig. S6** Self-activation toxicity test of bait protein *VaPYL9* and prey protein *VvPCMT*. Dental plaque didn’t show blue. They were verified that there were no self-activation toxicity.


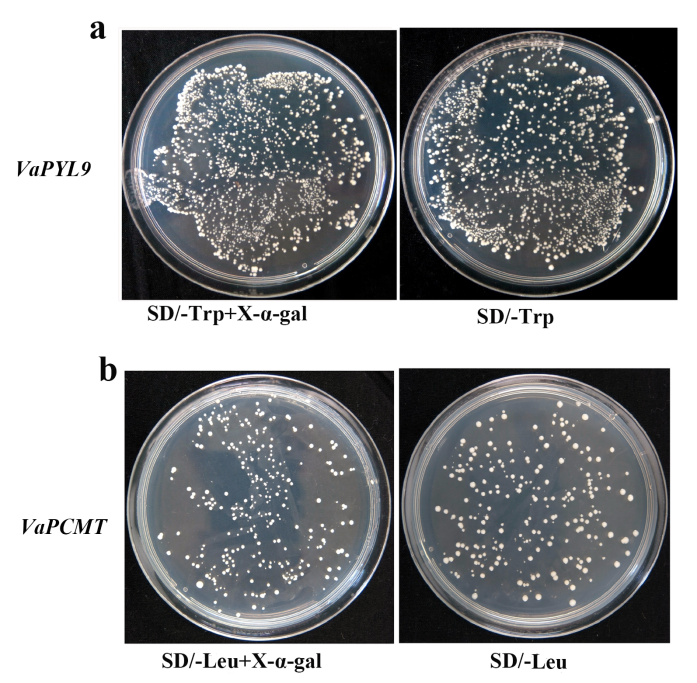

Supplement: Supplementary file 8 — Additional file 8: Supplementary Fig S6. Self-activation toxicity test of bait protein VaPYL9 and prey protein VaPCMT. Dental plaque didn’t show blue. They were verified that there were no self-activation toxicity. [file 12870_2022_3704_MOESM8_ESM.docx]
