# Supplementary material for: Overexpression VaPYL9 improves cold tolerance in tomato by regulating key genes in hormone signaling and antioxidant enzyme
Source: BMC Plant Biol. 2022 Jul 15;22:344. doi: 10.1186/s12870-022-03704-8 (PMC9284830; doi:10.1186/s12870-022-03704-8)
Supplement: Supplementary file 9 — Additional file 9: Supplementary Fig S7. Co-transformation of VaPYL9 and VaPCMT. VaPYL9 and VaPCMT were together introduced into Y2H gold and its presence was identified in Y2H gold by 1 % agarose gel electrophoresis. [file 12870_2022_3704_MOESM9_ESM.docx]

Additional file 9

**Fig. S7** Co-transformation of *VaPYL9* and *VaPCMT. VaPYL9* and *VaPCMT* were together introduced into Y2H gold and its presence was identified in Y2H gold by 1% agarose gel electrophoresis.


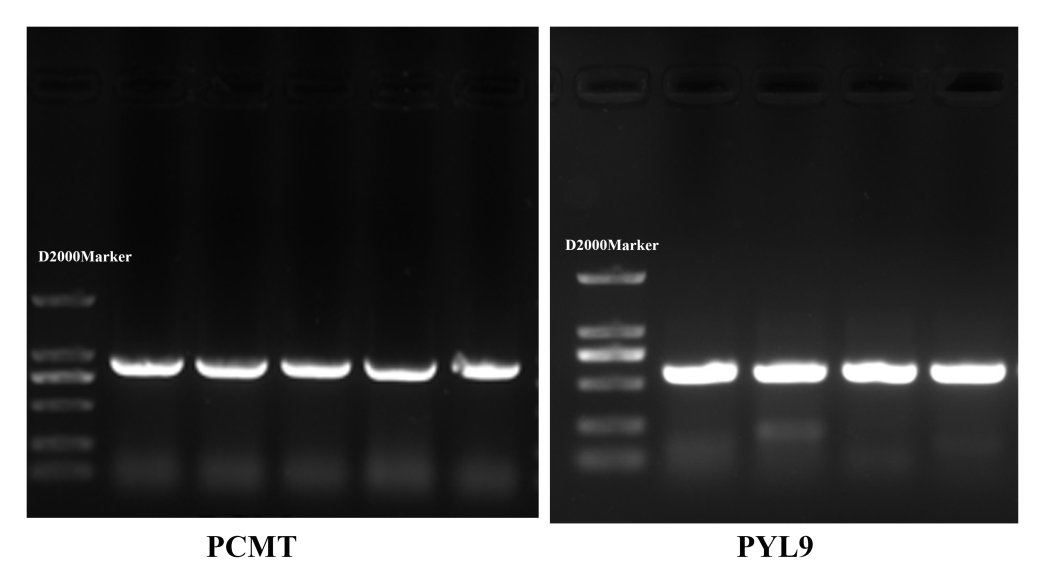


**a**

**b**
